# Combinatorial effects of cannabinoid receptor 1 and 2 agonists on characteristics and proteomic alteration in MDA-MB-231 breast cancer cells

**DOI:** 10.1371/journal.pone.0312851

**Published:** 2024-11-11

**Authors:** Chartinun Chutoe, Ingon Inson, Sucheewin Krobthong, Nut Phueakphud, Tueanjai Khunluck, Patompon Wongtrakoongate, Narattaphol Charoenphandhu, Kornkamon Lertsuwan

**Affiliations:** 1 Department of Biochemistry, Faculty of Science, Mahidol University, Bangkok, Thailand; 2 Center for Neuroscience, Faculty of Science, Mahidol University, Bangkok, Thailand; 3 Center of Calcium and Bone Research (COCAB), Faculty of Science, Mahidol University, Bangkok, Thailand; 4 HRH Princess Chulabhorn College of Medical Science, Chulabhorn Royal Academy, Bangkok, Thailand; 5 Department of Physiology, Faculty of Science, Mahidol University, Bangkok, Thailand; 6 Institute of Molecular Biosciences, Mahidol University, Nakhon Pathom, Thailand; 7 The Academy of Science, The Royal Society of Thailand, Bangkok, Thailand; Cinvestav-IPN, MEXICO

## Abstract

Breast cancer is the most common cancer diagnosed in women worldwide. However, the effective treatment for breast cancer progression is still being sought. The activation of cannabinoid receptor (CB) has been shown to negatively affect breast cancer cell survival. Our previous study also reported that breast cancer cells responded to various combinations of CB1 and CB2 agonists differently. Nonetheless, the mechanism underlying this effect and whether this phenomenon can be seen in other cancer characteristics remain unknown. Therefore, this study aims to further elucidate the effects of highly selective CB agonists and their combination on triple-negative breast cancer proliferation, cell cycle progression, invasion, lamellipodia formation as well as proteomic profile of MDA-MB-231 breast cancer cells. The presence of CB agonists, specifically a 2:1 (ACEA: GW405833) combination, prominently inhibited colony formation and induced the S-phase cell cycle arrest in MDA-MB-231 cells. Furthermore, cell invasion ability and lamellipodia formation of MDA-MB-231 were also attenuated by the exposure of CB agonists and their 2:1 combination ratio. Our proteomic analysis revealed proteomic profile alteration in MDA-MB-231 upon CB exposure that potentially led to breast cancer suppression, such as ZPR1/SHC1/MAPK-mediated cell proliferation and AXL/VAV2/RAC1-mediated cell motility pathways. Our findings showed that selective CB agonists and their combination suppressed breast cancer characteristics in MDA-MB-231 cells. The exposure of CB agonists also altered the proteomic profile of MDA-MB-231, which could lead to cell proliferation and motility suppression.

## Introduction

Endocannabinoid system (ECS) is an endogenous signaling system that regulates a wide range of biological processes in organisms such as immune response, brain development and bone metabolism, etc. [1–4]. ECS system consists of 3 main components including cannabinoid receptors (CB), endogenous cannabinoids (eCBs), and the enzymes for eCB synthesis and degradation [4]. There are 2 types of canonical CBs namely cannabinoid receptor 1 (CB1) and cannabinoid receptor 2 (CB2), both of which express throughout human body [5]. These receptors are activated by their agonists, such as anandamide (AEA) and 2-arachimoylglycerol (2-AG), the most well-known eCBs in humans. While eCBs were produced primarily by nerve cells, this could result in different combination in eCBs presence in each organ innervated by them [6, 7].

Besides its essential roles in maintaining many cellular processes, ECS was shown to have a potential protective role against cancer progression. Both CB1 and CB2 were expressed and influenced cell metabolism in many types of cancer including breast cancer [8, 9]. The presence of CB agonists hindered breast cancer cell survival and migration, and the exposure of various combinations of CB1 and CB2 agonists were shown to have different impacts on breast cancer cell behaviors [10]. Furthermore, the presence of CB2-selective agonist was shown to disrupt breast cancer-bone cell interaction and decrease breast cancer-induced bone remodeling in both *in vitro* and *in vivo* [10, 11]. The activation of ECS was shown to affect several cell survival pathways in breast cancer, such as epidermal growth factor (EGF), insulin-like growth factor-1 (IGF-1), casepase3 and nuclear factor kappa B (NF-κB) p65 [10, 12, 13]. Regardless, how different combinations of CB1 and CB2 agonists affect cellular signaling pathways in breast cancer is not known.

As previous study indicated the negative effects of specific combination of CB agonists on cancer cell viability, whether this mechanism involves cell cycle regulation is not known. In addition to uncontrollable cell proliferation, breast cancer recurrence is one of the critical problems in cancer treatment. This involved the retention of rapid self-renewal of cancer stemness, which is essential for both primary tumor proliferation and metastatic colonization [14–16]. However, whether the exposure of CB agonists could affect breast cancer cell growth and colony formation even after treatment removal is yet to be elucidated. Furthermore, cancer metastasis happening in later stages in cancer progression also possess a great challenge in cancer therapy. This mechanism involves the initial epithelial-to-mesenchymal transition (EMT), resulting in the formation of cellular lamellipodia to facilitate cancer cell movement and invasion [17, 18]. Previous report showed that CB activation suppressed breast cancer cell migration [10]. However, the effect of CB agonist exposure on cancer invasion through extracellular matrix, which is another key mechanism facilitating cancer metastasis [19], is not known. Herein, effects of CB1 and CB2 agonists and their combination on cancer progression-related behaviors including colony formation, invasion and cellular lamellipodia formation were investigated. Moreover, a high throughput proteomic approach was utilized to broaden the understanding and reveal the potential signaling pathways, by which CBs suppressed MDA-MB-231 breast cancer characteristics.

## Materials and methods

### Cell culture

In this study, MDA-MB-231 (RRID CVCL_0062; HTB-26, ATCC, VA, USA) was used as a human triple-negative breast cancer cell model; while, MCF-7 (RRID CVCL_0031; HTB-26, ATCC) was used as a control less aggressive breast cancer model. The cells were maintained in Dulbecco’s modified Eagle’s medium (DMEM) (12800017, Gibco, Texas, USA) supplemented by 10% fetal bovine serum (F7524, Sigma-Aldrich, MO, USA) and 1% penicillin-streptomycin (15140122, Gibco, Texas, USA). Moreover, 4T1 (RRID CVCL_0125; CRL-2539, ATCC) was used as a mouse triple-negative breast cancer cells, which were maintained in RPMI 1640 medium (Gibco) supplemented 10% fetal bovine serum (Sigma-Aldrich), 1% Sodium pyruvate (11360070, Gibco) and 1% penicillin-streptomycin (Gibco). The cells were maintained at 37°C with 5% CO_2_ in a humidified incubator and routinely tested for mycoplasma contamination by using the MycoStrip™ (Mycoplasma detection kit; rep-mys-50, InvivoGen, CA, USA).

### Chemicals

CB receptor agonists ACEA (A9719, Sigma-Aldrich) and GW405833 (G1421, Sigma-Aldrich) were used in this study. Since ACEA has K_i (CB1)_/ K_i (CB2)_ = 1,428-fold and GW405833 has K_i (CB2)_/ K_i (CB1)_ = 1,217-fold [20, 21]; therefore, ACEA and GW405833 represented the highly selective CB receptor agonists to CB1 and CB2 in our study, respectively. Since both agents were dissolved in DMSO, DMSO was used as vehicle control for all experiments. The concentration of CB agonists used in this study included 7.5 *μ*M ACEA, 15 *μ*M ACEA, 30 *μ*M ACEA, 15 *μ*M GW405833, 1:1 combination (15 *μ*M ACEA: 15 *μ*M GW405833), 1:2 combination (7.5 *μ*M ACEA: 15 *μ*M GW405833) and 2:1 combination (30 *μ*M: 15 *μ*M GW405833).

### Cell viability assay

The cytotoxic effect of CB agonists was carried out by cell viability assay as previously described [10]. Briefly, MDA-MB-231, MCF-7 and 4T1 cells were seeded in 96-well tissue culture plate (Jet Bio-Fil, Guangzhou, China) at a density of 1.0×10^4^ cells/well. After 24 hours of incubation, cells were treated with CB agonists, ACEA and/or GW405833, for 48 hours. The cell viability was investigated by using MTT assay. The experiment was carried out in three independent biological replicates with three technical replicates each.

### Clonogenic assay

MDA-MB-231 and MCF-7 cells were seeded in 6-well tissue culture plate (Jet Bio-Fil) at a density of 3.3×10^5^ cells/well. After incubation for 24 hours, the cells were treated with CB agonists for 48 hours. Subsequently, cells were harvested by trypsinization (Trypsin, 25200072, Gibco, Texas, USA) and seeded into the new 6-well plate at a density of 800 cells/well. Then, the cells were maintained in complete culture medium, and the medium was replaced every two days. After 12 days of incubation, all cell culture medium was discarded followed by two PBS washes. The cells were fixed by 100% methanol (106009, Sigma-Aldrich, PA, USA) for 30 minutes. Colonies were stained with a staining solution, 0.5% crystal violet (V5265, Sigma-Aldrich) in 0.25% methanol. After staining, scanned images were processed using ImageJ software (National Institutes of Health (NIH), MD, USA). To distinguish separated colony area, all images were analyzed through colony area plugin for ImageJ developed by Guzmán et al. [22]. The processed images were then adjusted to separate nearby colonies by using the watershed binary. To count the numbers of colonies, colony numbers were measured by using analyzed particles tool in ImageJ software. The clonogenic assay was performed in at least three independent biological replicates with two internal technical replicates each.

### Cell cycle analysis

The percentage of cells in each cell cycle phase was determined using modified method from Lertsuwan *et*. *al*., [23]. Briefly, MDA-MB-231 cells were seeded in 6-well tissue culture plate (Jet Bio-Fil) at a density of 3.3×10^5^ cells/well for 24 hours. Then, they were treated with ACEA, GW405833 or the combination of ACEA and GW405833 in different ratios for 48 hours. After that, the cells were collected by trypsinization and fixed in cold 70% ethanol (A6531-04, Fulltime, China) at –20 ˚C. Afterwards, the samples were centrifuged for 5 minutes at 2,500 rpm and treated with 20 *μ*g/mL propidium iodide (PI) DNA staining solution (P1304MP, Life Technologies, CA, USA) and 200 *μ*g/mL PureLink™ RNase A (12091021, Life Technologies) for 30 minutes at room temperature in the dark. Cell cycle distribution was analyzed using a Flow Cytometer (Accuri™ C6 Plus; BD Biosciences, CA, USA). The experiment was performed in four independent biological replicates.

### Sample preparation for proteomic analysis

MDA-MB-231 cells were plated at 3.3×10^5^ cells/well in 6-well tissue culture plate (Jet Bio-Fil) and allowed to attach overnight. The cells were then treated with ACEA, GW405833 or the 2:1 combination of both agents for 48 hours. At the end of the experiment, cell pellets were collected and were processed using a modified protocol based on a previously published proteome-profiling pipeline [24, 25]. Briefly, cell pellets were washed twice with PBS supplemented with protease inhibitor cocktail (04693159001, Roche, Mannheim, Germany). Afterwards, total proteins were extracted using modified radioimmunoassay precipitation (RIPA) buffer containing the protease inhibitor cocktail for 60 minutes at 4°C. Then, samples were centrifuged at 12,000 rpm for 30 minutes, and protein-containing supernatants were collected. Protein concentration of samples was measured by using BCA protein assay kit (23227, Thermo Scientific). In this study, protein samples from 3 independent experiments were used for further analysis.

To investigate protein expression profile in MDA-MB-231 cells exposed to CB agonists, extracted proteins were precipitated using ice-cold acetone. After precipitation, the protein pellet was reconstituted and adjusted to 1 *μ*g/*μ*L in reconstitution buffer, 0.2% RapiGest SF (186001860, Waters, UK) in 10 mM ammonium bicarbonate. To carry out sulfhydryl alkylation, fifty microliters of the protein solution (50 *μ*g of proteins) were mixed with 5 mM DTT in 10 mM ammonium bicarbonate for 20 minutes at 90°C followed by incubation at room temperature for 40 minutes in the dark. After that, the mixture was placed in a desalting column (89883, Zebra-spin, Thermo Scientific) with trypsin at a ratio of 1:50 (enzyme: protein) to digest the peptide at 37°C. After 6 hours of incubation, 0.2% formic acid was added at a 1:1 (v/v) ratio to terminate the digestion process. The peptides were reconstituted in 0.1% formic acid and transferred to TruView LCMS vials (186005666CV, Waters, UK) for further analysis.

### LC-MS/MS setting

The tryptic peptides were analyzed using LC-MS instrument consisting of an EASY-nLC 1200 system with a Q Exactive HF Orbitrap (Thermo Scientific). One microgram of peptides was separated on EASY-SPRAY PEPMAP RSLC C18 column (25 cm length x 75 *μ*m inner diameter, 2 *μ*m particle size (ES802, Thermo Scientific)). The flow rate was set at 300 nL/min with mobile phase A (0.1% formic acid in LC-MS grade water) and mobile phase B (0.1% in LC-MS grade acetonitrile). The column was maintained at 60°C. The linear gradient was changed from 2% to 45% of mobile phase B in 75 minutes and held for 15 minutes. The gradients were then increased to 95% of mobile phase B for 5 minutes followed by re-equilibration to 2% mobile phase B for additional 20 minutes. For MS1, the scanning range was set at 400–1,650 *m/z* with a resolution of 120,000, 3×10^6^ automated grain control (AGC) and 50 ms injection time (IT). For MS2, the resolution was set at 15,000 with 1×10^5^ AGC and 25 ms IT. The data was normalized to the higher energy collisional dissociation (HCD) fragmentation of 28 and processed by Proteome Discoverer 2.4 against the UniProt protein database for *Homo sapiens*. For protein and peptide identification, the parameters were set as follows: 1% of false discovery rates (FDR); peptide tolerance = 20 ppm; fragment tolerance = 0.05 Da; minimum fragment ion matches per peptide = 3; digest enzyme = trypsin; fixed modification = cysteine carbamidomethylation; variable modification = methionine oxidation. The normalization of relative protein abundance ratio was performed using the total peptide amount of each LC-runs (across all proteomic samples n = 24; 8 conditions with biological triplicate each). The proteomics data including raw files and search results have been deposited to the ProteomeXchange Consortium via the PRIDE partner repository with the dataset identifier PXD040666.

### Data analysis, bioinformatic analysis and meta-analysis

Data analysis of proteomic profile from MDA-MB-231 exposed to CB agonists was performed by using Reactome (reactome.org) [26]. Then, the proteins with statistical significance (*p* < 0.01) were processed to gene ontology enrichment analysis by using Panther (pantherdb.org) [27]. Differential expression of proteins from each treatment as compared to control was reported as log_2_ fold change normalized to control.

After that, protein-protein interaction (PPI) analysis was performed through STRING (string-db.org) [28] to observe the protein relationship and possible affected pathway. Furthermore, all PPI networks were constructed and visualized by using Cytoscape 3.9.1 software (cytoscape.org) [29].

For the meta-analysis study of gene expression, the analyses were acquired by using GEPIA2 (gepia2.cancer-pku.cn) [30]. In addition, to analyze the expression profile of breast cancer, the 179 datasets were obtained from the Cancer Genome Atlas Program (TCGA) and the Genotype-Tissue Expression (GTEx) databases for each breast cancer subtype in comparison to normal tissues. According to breast cancer subtypes, they are categorized based on their mutation and expression profiles into 4 molecular subtypes including luminal A (ER+, PR+ ≥ 20%, HER2− and Ki67+ < 20%), luminal B (ER+, PR+ < 20%, HER2− or HER2+, Ki67+ ≥ 20%), non-luminal (ER-, PR-, overexpressed HER2) and basal-like, namely triple-negative breast cancer (TNBC), (ER−, PR−, HER2−) [31, 32]. The statistical analysis was carried out by using analysis of variance (ANOVA).

### Transwell invasion assay

Before cell seeding, the membrane of upper chamber was coated with diluted Matrigel (356234, Corning, USA) at a ratio of 1:5 with serum-free culture medium. MDA-MB-231 cells were seeded in serum-free culture media at 5×10^4^ cells per well on an 8 *μ*m pore size Transwell insert (Falcon, Arizona, USA). In the lower chamber, DMEM culture medium supplemented with 20% FBS was added. Cells were treated with either 30 *μ*M ACEA, 15 *μ*M GW405833 or their 2:1 combination. Then, cells were cultured at 37°C with 5% CO_2_ for 12 hours before staining with 0.5% crystal violet at room temperature. Pictures of invasive cells on the underside of the membrane were taken under a light microscope (Model MBL3200, A. KRÜSS Optronic GmbH, Hamburg, Germany). After that, the number of invasive cells was quantified using ImageJ software (NIH). The quantification of invasive cells was carried out following the previous published protocol [33]. All images were converted into 8-bit format, and the nearby invasive cells were separated by watershed binary tool. The numbers of invasive cells were counted by analyzed particles tool. Invasion assay was carried out for four independent biological replicates.

### Lamellipodia formation assay

MDA-MB-231 cells were seeded into 8-well glass bottom chamber (LAB-TEK, New York, USA) at a density of 1×10^4^ cells per chamber. After 24 hours of seeding, 30 *μ*M ACEA, 15 *μ*M GW405833 or their 2:1 combination were applied to MDA-MB-231 cells for 12 hours. Then, cells were fixed with 4% formaldehyde (20909.330, VWR international, Rontenay-Sous-Bois, France) before staining with Alexa Fluor™ 546 Phalloidin (A22283, Thermo Scientific, MA, USA) for F-actin staining and Hoechst (62249, Thermo Scientific) for nuclei staining. Filopodia and lamellipodia formation was observed using confocal laser scanning microscope (FV1000, Olympus, Tokyo, Japan). Image analysis was performed using ImageJ software (NIH). To perform the measurement, all images were converted into 8-bit format. The quantification was carried out by using modified protocol from Weaver et. al. [34]. The polygon selection tool was used to select the edge of individual cells and to measure the whole cell spread in *μ*m^2^ according to scale bar. Whole cell spread was the average surface area of individual cells. Experiments were performed in six independent biological replicates.

### Quantitative reverse transcription polymerase chain reaction (qRT-PCR)

To validate the proteomic results, the expression of representative genes in each figure was evaluated by qRT-PCR. MDA-MB-231 cells were seeded at a density of 3.3×10^5^ cells/well into 6-well plates (Jet Bio-Fil). Treatments including vehicle control (DMSO), 30 *μ*M ACEA, 15 *μ*M GW405833, and the 2:1 combination (30 *μ*M ACEA and 15 *μ*M GW405833) were applied to MDA-MB-231 cells after 24 hours of seeding. MDA-MB-231 cells were collected 48 hours post-treatment, and their RNA was extracted using RNeasy Mini Kit (74104, Qiagen, Hilden, Germany). One microgram of RNA was converted into cDNA by using iScript™ (1708841, Bio-Rad, CA, USA). Thereafter, 100 nanograms of cDNA were combined with primers specified in the S1 Table and Luna® Universal qPCR Master Mix (M3003S, Biolabs, Herts, UK). After that, the mixtures were added to qPCR microstrips (TLS0801, Bio-Rad), and qRT-PCR was performed utilizing the Bio-rad CFX connect™ Real-Time PCR Detection System. The relative expression was quantified from Ct value normalized to β-actin of their own samples. Experiments were performed in three independent biological replicates.

### Statistical analysis

Statistical analysis of all results was performed by using GraphPad Prism 9 (GraphPad Software Inc., USA). The comparison between 2 groups was performed using a T-test. For the multiple comparisons among treatment groups, the results were analyzed by using one-way ANOVA. Then, Tukey post-test was carried out to determine the statistical difference between pairs of means. For all statistical analysis, *p-*value ≤ 0.05 was considered as statistical significance.

## Results

### Pre-exposure of specific combination of ACEA and GW405833 suppressed MDA‑MB‑231 colony formation

**Fig 1:**
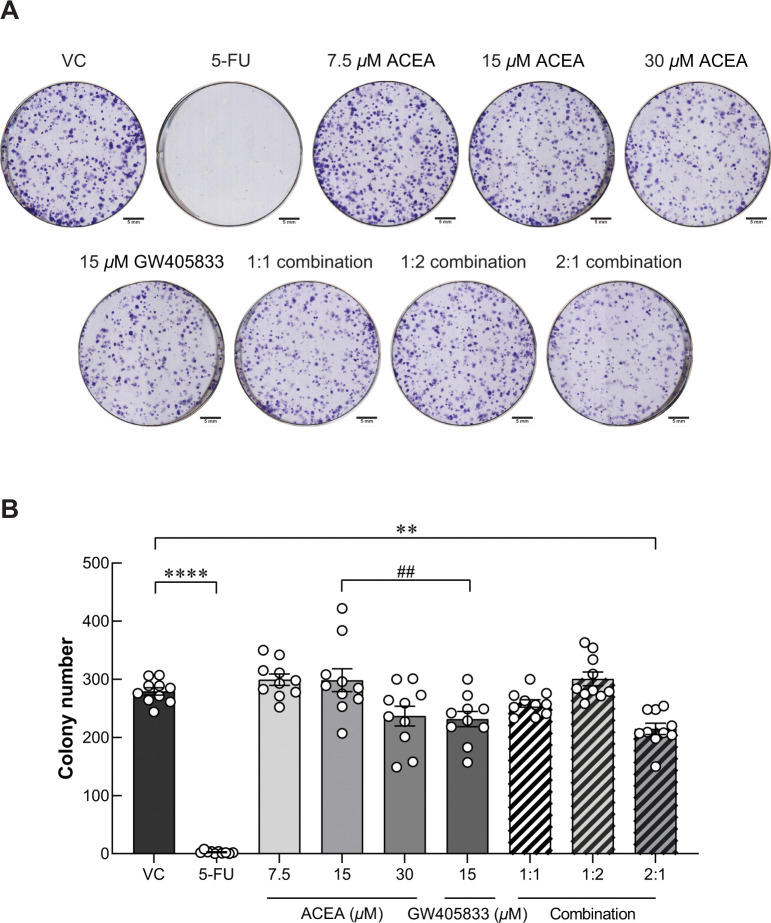
A specific combination of ACEA and GW405833 shows long-term inhibition on MDA-MB-231 colony formation. (A) Representative images of colony formation of MDA-MB-231 pre-exposed to ACEA, GW405833 and their combination; (B) Colony number of MDA-MB-231 pre-exposed to CB agonists was represented by mean ± SEM from five independent biological replicates with internal technical duplicates each. (**p < 0.01, ****p < 0.0001, while ##*p* < 0.01).

According to our previous study, ACEA, GW405833 and their combination at different ratios suppressed MDA-MB-231 cell viability by apoptosis induction, especially in the 2:1 combination of ACEA and GW405833 [10]. In this study, cytotoxicity of CB agonist treatment was also investigated in the less aggressive human breast cancer cells (MCF-7) and mouse triple-negative breast cancer cells (4T1) as shown in S1 Fig. Our results showed that MCF-7 was notably less sensitive to CB agonists as compared to MDA-MB-231. A specific 2:1 combination ratio also showed a significant cytotoxic effect on both human and mouse triple-negative breast cancer cell lines but not for MCF-7 (S1I Fig). However, whether the growth inhibitory effects would still persist in the surviving cells after the treatment withdrawal is not known. CB agonist concentrations were determined by cell viability test as reported in our previously published data [10]. Accordingly, the same concentrations were used in this study. The long-term suppression was then examined by clonogenic assay in this study. After MDA-MB-231 cells were pre-exposed to ACEA and GW405833 at different concentrations and combination ratios including vehicle control, 7.5*μ*M ACEA, 15 *μ*M ACEA, 30*μ*M ACEA, 15*μ*M GW405833, 1:1 combination (15 *μ*M ACEA and 15 *μ*M GW405833), 1:2 combination (7.5 *μ*M ACEA and 15 *μ*M GW405833) and 2:1 combination (30 *μ*M ACEA and 15 *μ*M GW405833), surviving cells were seeded into the new plate for colony formation assay. The pre-exposure of 100 *μ*g/mL (768.76 *μ*M) of 5-Fluorouracil (5-FU) was used as positive control, which completely inhibited MDA-MB-231 colony formation even after treatment withdrawal for 12 days. Our results showed that MDA-MB-231 cells were more sensitive to GW405833 than ACEA as shown in Fig 1. At the same concentration of 15 *μ*M, MDA-MB-231 colony formation was significantly lowered in GW405833 treated cells than ACEA treated group. As none of individual treatments significantly decreased colony number as compared to control, our results showed that only the combination at 2:1 ratio (30 *μ*M ACEA and 15 *μ*M GW405833) significantly reduced the number of MDA-MB-231 colony formation by 23.14% as compared to control (Fig 1A and 1B). Nevertheless, the 2:1 combination of ACEA and GW405833 did not affect the colony formation in MCF-7 cells (S2A and S2B Fig). The findings indicated that the 2:1 combination of ACEA and GW405833 promoted long-term suppression of MDA-MB-231 colony formation, while individual treatments and other combinations did not.

### Co-activation of CB receptors induced cell cycle arrest in MDA‑MB‑231

**Fig 2:**
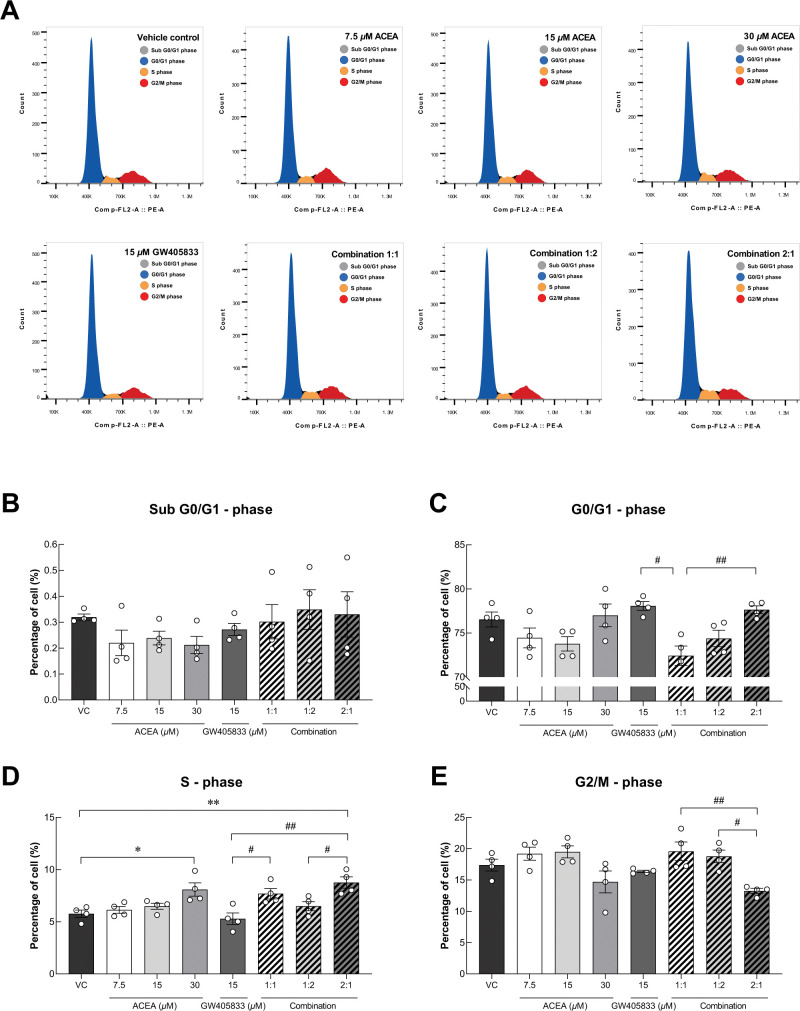
MDA-MB-231 cell cycle distribution after CB agonist treatment were evaluated. (A) Scatter plot of cells in different cell cycle phases stained with 20 *μ*g/mL PI after 48 hours of CB agonist treatment. Quantitative results of cell distribution in each cell cycle phases (B) Sub G0/G1 phase, (C) G0/G1 phase, (D) S phase, (E) G2/M phase were presented as mean ± SEM from four independent biological replicates. (*p < 0.05, **p < 0.01, while #*p* < 0.05, ##*p* < 0.01).

As shown in Fig 1, a specific combination of CB agonists suppressed colony formation ability of MDA-MB-231 cells. To further study whether cell cycle alteration involved in CB-induced growth inhibition in breast cancer cells (MDA-MB-231) seen in previous and current studies, flow cytometry was carried out to investigate changes in cell cycle progression as shown in representative histograms in Fig 2A. Our results showed that there was no significant difference of cells in Sub-G0/G1, G0/G1 and G2/M phases between treatments as compared to control (Fig 2B, 2C and 2E). However, the proportion of cells in S-phase was markedly increased in MDA-MB-231 exposed to 30 *μ*M ACEA and the 2:1 (ACEA: GW405833) combination by 33.51% and by 41.18% as compared to control, respectively (Fig 2D). Moreover, this combination also significantly augmented the percentage of cells in S-phase than the individual treatment of 15 *μ*M GW405833. In addition, the 2:1 combination was shown to have the lowest proportion of MDA-MB-231 cells in the G2/M phase as compared to other combination treatments (Fig 2E). These results suggested that colony formation suppression in 2:1 combination corresponded to S-phase cell cycle arrest.

### Proteomic profile alteration of proteins related to cell proliferation and survival of MDA-MB-231

**Fig 3:**
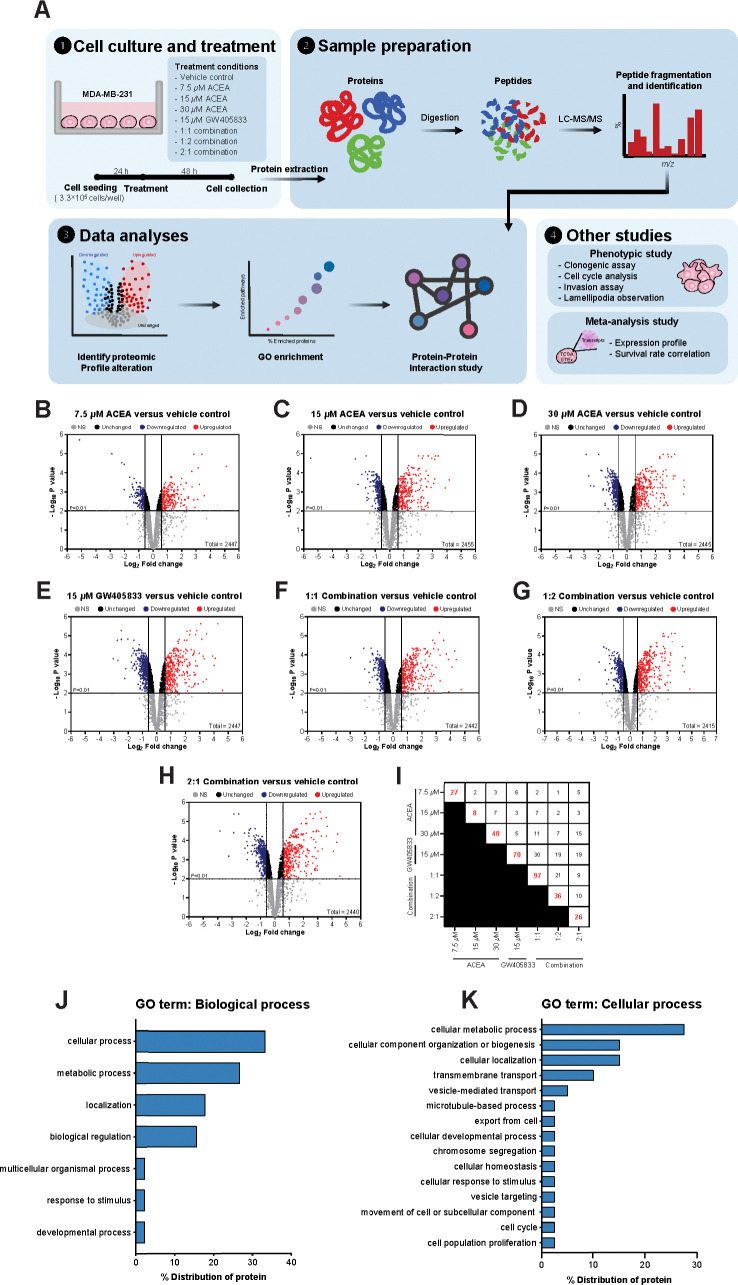
Proteomic analyzed signature profiles of MDA-MB-231 exposed to different combinations of CB agonists. (A) The schematic workflow of proteomic profile study of MDA-MB-231 treated with CB agonists. Volcano plot of differential protein expression shows a negative base 10 log of the adjust *p-*values plotting against the base 2 log of the fold change of each protein in CB agonists treated group normalized to control, (B) 7.5 *μ*M ACEA; (C) 15 *μ*M ACEA; (D) 30 *μ*M ACEA; (E) 15 *μ*M GW405833; (F) 1:1 combination; (G) 1:2 combination; (H) 2:1 combination, versus vehicle control group. (I) Chart showed the unique proteins after exposed to CB treatments. The GO term enrichment analysis (J) biological process and (K) cellular process.

**Fig 4:**
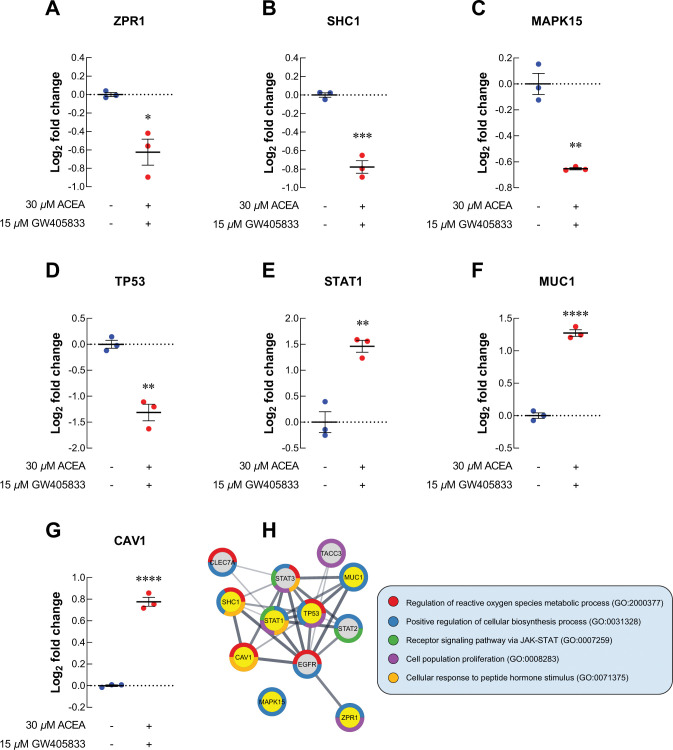
Proteomics study revealed the alteration of uniquely related proteins to cell proliferation in MDA-MB-231. The level of cell cycle and proliferation related proteins after exposed to 2:1 combination treatment expressed by log_2_ fold change of each protein in CB agonists treated group normalized to control (A) ZPR1; (B) SHC1; (C) MAPK15; (D) TP53; (E) STAT1; (F) MUC1; (G) CAV1; (H) Protein-protein interaction diagram of changed cell proliferation-related proteins in MDA-MB-231 treated 2:1 combination. The connected lines indicated the potential direct associations of proteins with the bolder lines represented the stronger interactions. All dot plots were presented as individual log_2_ fold change ± SEM of three biological replicates. (*p < 0.05, **p < 0.01, ***p < 0.001, ****p < 0.0001).

To identify the proteomic signatures that were altered in breast cancer cells exposed to CB agonists, protein expression profile of MDA-MB-231 cells exposed to vehicle control, CB agonists alone or their different combination was determined using LC-MS/MS. The schematic workflow of proteomic study in this research was shown in Fig 3A. Firstly, the differential protein expression analysis was performed between each treatment group against vehicle control using Reactome (reactome.org), and the proteins with *p*-values lower than 0.01 and more than 1.5-fold-change were shown in volcano plots in Fig 3B–3H. The numbers of significantly downregulated and upregulated proteins as compared to vehicle control were shown in Table 1. Among those, the top 5 downregulated and upregulated proteins from each treatment as compared to control were shown in Table 2, and the numbers of unique proteins that were significantly altered in each individual and combination treatment were shown in Fig 3I (in red). Since the 2:1 (ACEA:GW405833) combination was shown to have the most significant effects on MDA-MB-231 cell growth in previous and current studies, proteomic signature in this group was further analyzed. The results showed that there were 26 unique proteins altered in the 2:1 combination treatment as compared to other treatments (S2 Table and Fig 3I). We next performed an enrichment analysis of gene ontology on these unique proteins using Panther (pantherdb.org). Our results indicated that 15 unique proteins in 2:1 combination treatment were involved in cellular processes (Fig 3J). Within these 15 unique changed proteins, one protein related to cell population proliferation process (Zinc Finger Protein; ZPR1) and one protein involved in cell cycle (Anaphase Promoting Complex Subunit 1; ANAPC1) were identified (Fig 3K). For proliferation-related protein, we found that ZPR1 was significantly decreased by 1.54-fold in MDA-MB-231 treated with the 2:1 combination of CB agonists as compared to a vehicle control (Fig 4A). Considering all significantly changed proteins in MDA-MB-231 exposed to the 2:1 combination as compared to control, other cell survival and proliferation-related proteins were also detected. SHC-transforming protein 1 (SHC1), mitogen-activated protein kinase 15 (MAPK15) and tumor protein P53 (TP53) were downregulated by 1.71-, 1.57- and 2.49-fold upon the exposure of this combination, respectively (Fig 4B–4D). Gene expression validation of ZPR1, SHC1, MAPK15 and TP53 in MDA-MB-231 cells exposed to CB agonists was also shown by qRT-PCR (S3A–S3D Fig). On the other hand, our results showed that the exposure of the 2:1 combination induced the expression of signal transducer and activator of transcription 1 (STAT1), mucin 1 (MUC1) and caveolin 1 (CAV1) by 2.75-, 2.42- and 1.71-fold, respectively (Fig 4E–4G).

**Table 1:** The number of downregulated and upregulated proteins in each condition from volcano plots that is presented in Fig 3.

Treatments	Number of proteins found	
	Downregulate	Upregulated
7.5 *μ*M ACEA	196	242
15 *μ*M ACEA	272	301
30 *μ*M ACEA	338	301
15 *μ*M GW405833	434	329
1:1 Combination	362	341
1:2 Combination	341	357
2:1 Combination	397	355

**Table 2:** The top 5 downregulated and upregulated proteins of MDA-MB-231 cells after CB agonist exposure.

Conditions	Upregulated proteins		Downregulated proteins	
	Accession	Log_2_ FC	Accession	Log_2_ FC
7.5 *μ*M ACEA	KRT2	5.105	BIRC6	-5.151
	ALDOC	3.568	MTF2	-2.886
	GOLPH3	3.409	COMMD9	-2.198
	MBD3	3.232	DNAJB4	-2.022
	F3	3.161	FN1	-2.019
15 *μ*M ACEA	RCN2	4.159	CCNB1	-5.504
	ALDOC	3.899	SUPT7L	-2.386
	GOLPH3	3.398	MAGI1	-2.214
	SMC3	3.193	H3-3A	-1.942
	MBD3	3.147	H4C1	-1.681
30 *μ*M ACEA	ALDOC	4.014	SUPT7L	-2.854
	RCN2	3.983	MAGI1	-2.681
	ARHGAP18	3.957	LCN2	-2.540
	SMC3	3.365	MT1G	-2.306
	GOLPH3	3.339	RBM10	-1.968
15 *μ*M GW405833	EIF4H	4.598	MAGI1	-3.050
	SMC3	4.275	NOSIP	-2.755
	RCN2	4.095	MT1G	-2.715
	ALDOC	4.018	RBM10	-2.490
	JPT1	3.920	SUPT7L	-2.463
**1:1 Combination**	EIF4H	4.738	MAGI1	-2.984
	RCN2	4.401	RBM10	-2.058
	KRT2	4.286	CPSF7	-1.993
	ALDOC	4.112	SRF	-1.694
	LRRFIP1	3.882	HIP1R	-1.654
**1:2 Combination**	EIF4H	4.833	MAGI1	-4.171
	RCN2	4.472	NOSIP	-2.557
	ARHGAP18	4.401	MTF2	-2.181
	ALDOC	4.264	CPSF7	-2.057
	SMC3	4.236	RBM10	-1.972
**2:1 Combination**	EIF4H	4.551	MAGI1	-3.836
	KRT2	4.390	MT1G	-3.222
	RCN2	4.336	NOSIP	-2.883
	ALDOC	4.096	SUPT7L	-2.860
	SMC3	3.674	MTF2	-2.524

**Fig 5:**
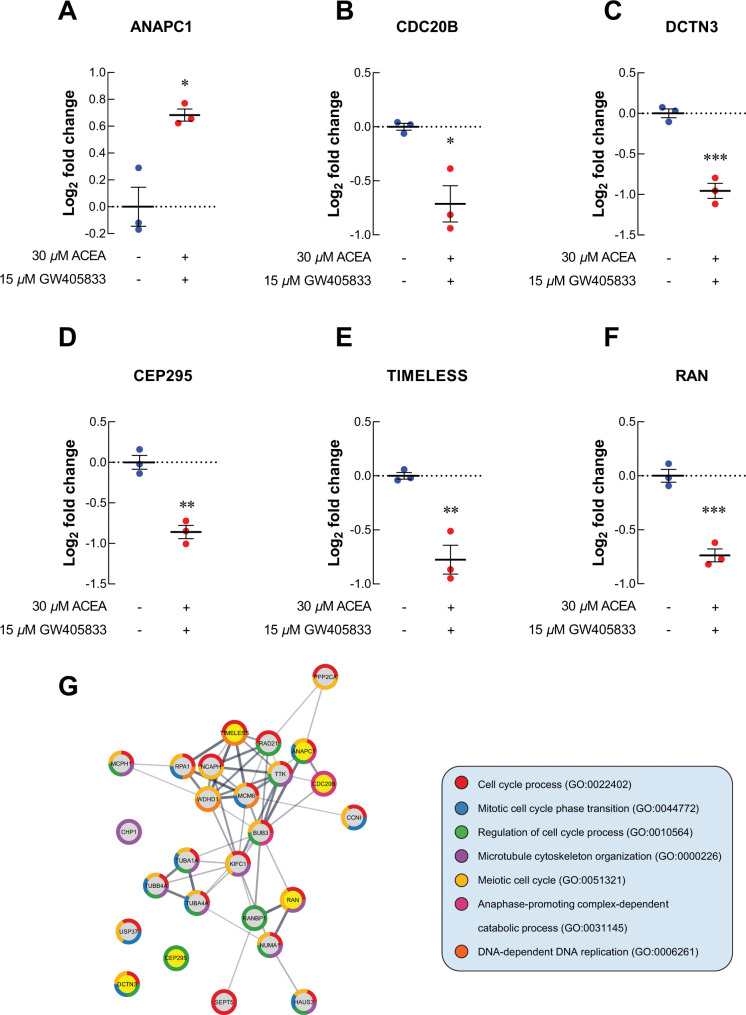
Proteomics study revealed the alteration of uniquely related proteins to cell cycle in MDA-MB-231. The level of cell cycle-related proteins after exposed to CB agonists and their combination treatment expressed as log_2_ fold change normalized to control (A) ANAPC1; (B) CDC20B; (C) DCTN3; (D) CEP295; (E) TIMELESS; (F) RAN; (G) Protein-protein interaction diagram of changed proteins in MDA-MB-231 exposed to CB agonists. The connected lines indicated the potential direct associations of proteins with the bolder lines represented the stronger interactions. All dot plots were presented as individual log_2_ fold change ± SEM of three biological replicates. (*p < 0.05, **p < 0.01, ***p < 0.001).

For cell cycle-related proteins, ANAPC1, a regulator of metaphase-to-anaphase transition, was upregulated in MDA-MB-231 exposed to 2:1 combination of ACEA and GW405833 as compared to vehicle control (Fig 5A). The increase in ANAPC1 expression in MDA-MB-231 cells exposed to CB agonists was also confirmed by qRT-PCR (S3E Fig). On the other hand, cell division cycle protein 20 homolog B (CDC20B), dynactin subunit 3 (DCTN3), centrosomal protein 295 (CEP295), timeless circadian regulator (TIMELESS) and Ras-related nuclear protein (RAN) were significantly decreased by 1.64-, 1.94-, 1.81-, 1.71- and 1.67-fold upon the exposure of 2:1 CB agonists, respectively (Fig 5B–5F). As these proteins may also interact with other proliferation-related and cell cycle-related proteins presented in MDA-MB-231 treated with 2:1 combination treatment, we next investigated potential protein-protein interaction of all proteins with significant alterations (*p* < 0.01) in our samples through STRING analysis. The protein-protein interaction (PPI) diagram for cell proliferation-related proteins was shown in Fig 4H and cell cycle-related proteins were shown in Fig 5G. Together, these results demonstrated the potential regulatory proteins propensity for colony formation suppression and S-phase cell cycle arrest in MDA-MB-231 exposed to 2:1 (ACEA:GW405833) as compared to control.

### CB receptor activation suppressed MDA‑MB‑231 cell invasion

**Fig 6:**
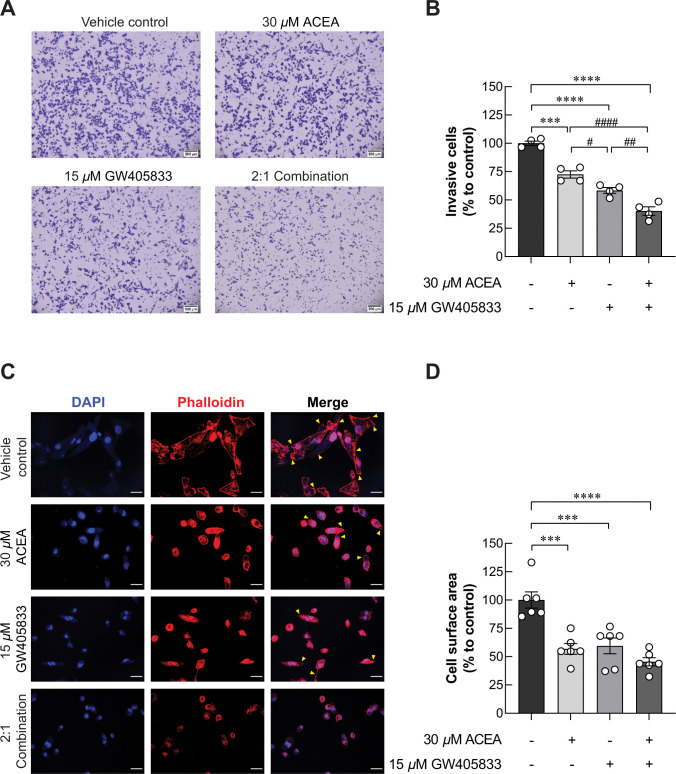
ACEA, GW405833 and their 2:1 combination inhibited MDA-MB-231 cell invasion and lamellipodia formation. (A) Representative images of MDA-MB-231 cell invasion after being treated for 12 hours. (B) Bar graph represented percentage of invasive cells as compared to control. (C) Representative images of whole cell spread and lamellipodium/filopodium formation (indicated in yellow arrows) in MDA-MB-231 after being treated for 12 hours. (D) Bar graph represented the percentage of whole cell spreading area. Each bar represents mean ± SEM of four independent biological replicates for transwell invasion assay and six independent biological replicates for lamellipodia formation assay. (***p < 0.001, ****p<0.0001, while #*p* < 0.05, ##*p* < 0.01, ####*p* < 0.0001).

Since the exposure of 2:1 (ACEA:GW405833) combination significantly reduced growth and promoted S-phase cell cycle arrest in MDA-MB-231, we aimed to test whether this condition also affected metastasis ability of MDA-MB-231 by conducting transwell invasion assay. Our results demonstrated that 30 *μ*M ACEA significantly reduced number of invasive cells by 27.45% as compared to control (Fig 6A and 6B). Besides, MDA-MB-231 cell invasion was also markedly reduced in 15 *μ*M GW405833 by 41.65% as compared to control. These results revealed the higher sensitivity of MDA-MB-231 cells to GW405833 than ACEA. Interestingly, combination between ACEA and GW405833 at ratio of 2:1 further suppressed MDA-MB-231 cell invasion by 59.80% as compared to control. Taken together, our results demonstrated that this specific combination was also shown to have significantly greater suppression of cell invasion as compared to individual treatments.

### Whole cell spreading and lamellipodia formation were reduced in MDA-MB-231 after being exposed to CB agonists

According to Fig 6A, 6B, MDA-MB-231 cell invasion capability was decreased from CB agonist exposure. Since the formation of lamellipodia is known as one of the key characteristics for migrating cells [35, 36], we then performed fluorescent staining of F-actin in MDA-MB-231 exposed to CB agonists. Our results revealed that filopodia and lamellipodia formation was significantly decreased in MDA-MB-231 exposed to CB agonist treatments as shown in Fig 6C. Whole cell spread area of MDA-MB-231 cells was considerably reduced by 43.18% and 40.52% in 30 *μ*M ACEA and 15 *μ*M GW405833, respectively. The most dramatic reduction was found in the 2:1 combination with the decreased whole cell spread area by 54.57% as compared to control (Fig 6D). These results showed that reduction of cell invasion-related morphology correspond to decreased cell invasion in CB agonist-treated cells.

### Quantitative proteomic analysis revealed the reduction of lamellipodia-promoting proteins; while, the induction of cell invasion suppressor proteins was observed upon CB agonist exposure

**Fig 7:**
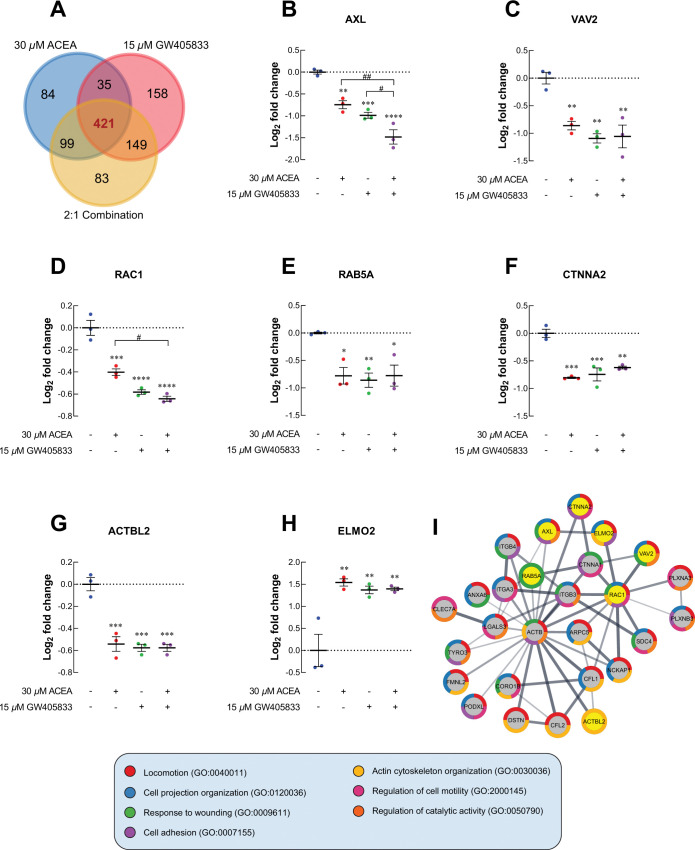
The cell locomotion-related proteomic signature profiles of MDA-MB-231 after exposed CB agonists were investigated. (A) Venn diagram comparing among 30 *μ*M ACEA, 15 *μ*M GW405833 and 2:1 combination. The dot plots represented protein expression as log_2_ fold change of CB agonists treated group normalized to control in MDA-MB-231;(B) AXL; (C) VAV2; (D) RAC1; (E) RAB5A; (F) CTNNA2; (G) ACTBL2; (H) ELMO2; (I) Protein-protein interaction diagram of cell locomotion related protein in 2:1 combination treatment. All dot plots were presented as individual log_2_ fold change ± SEM of three biological replicates. (*p < 0.05, **p < 0.01, ***p < 0.001, ****p < 0.0001 versus control, while #*p* < 0.05, ##*p* < 0.01).

Proteomic profile of MDA-MB-231 cells exposed to 30 *μ*M ACEA, 15 *μ*M GW405833 and their 2:1 combination was analyzed to compare the proteomic signature changes against vehicle control. Hence, proteins with statistical difference as compared to control were selected and analyzed. Among these altered proteins, we found that 421 proteins were commonly expressed in MDA-MB-231 cells exposed to these 3 conditions (Fig 7A). For proteins associated with cell locomotion, AXL receptor tyrosine kinase (AXL) was downregulated by 1.68-fold and 1.98-fold in MDA-MB-231 exposed to 30 *μ*M ACEA and 15 *μ*M GW405833, respectively. The 2:1 (ACEA:GW405833) combination ratio was found to further downregulate AXL protein by 2.79-fold as compared to control (Fig 7B). These CB agonist treatments significantly reduced the expression of cellular actin dynamic regulator, VAV2 guanine nucleotide exchange factor (VAV2), by 1.82, 2.13 and 2.08-fold, respectively (Fig 7C). Likewise, Rac family small GTPase 1 (RAC1) and Ras-related protein Rab-5A (Rab5A) were significantly downregulated in MDA-MB-231 cells after exposed to 2:1 (ACEA:GW405833) combination (Fig 7D and 7E). The decreased expression of AXL, VAV2 and RAC1 in MDA-MB-231 cells exposed to CB agonists was also confirmed by qRT-PCR as shown in S3F–S3H Fig. Additionally, proteins playing a role in cell-to-cell adhesion and actin polymerization, catenin alpha 2 (CTNNA2) and beta-actin-like protein 2 (ACTBL2) were downregulated in MDA-MB-231 exposed to CB agonists as shown in Fig 7D and 7G. On the other hand, engulfment cell motility 2 (ELMO2) was upregulated after CB agonist treatment (Fig 7H). Similar to Figs 4 and 5, cell locomotion-related proteins with significant change (*p* < 0.01) in 2:1 combination treatment were further analyzed for potential interaction by using STRING as shown as PPI diagram in Fig 7I. The PPI diagram illustrated the direct interaction among effected protein including VAV2, RAC1 and other lamellipodia-related proteins [37, 38]. Accordingly, our results suggested that CB agonists suppressed MDA-MB-231 cell invasion, and this effect was associated with lamellipodia formation suppression.

### ALDOC was upregulated in MDA-MB-231 exposed to CB agonists

**Fig 8:**
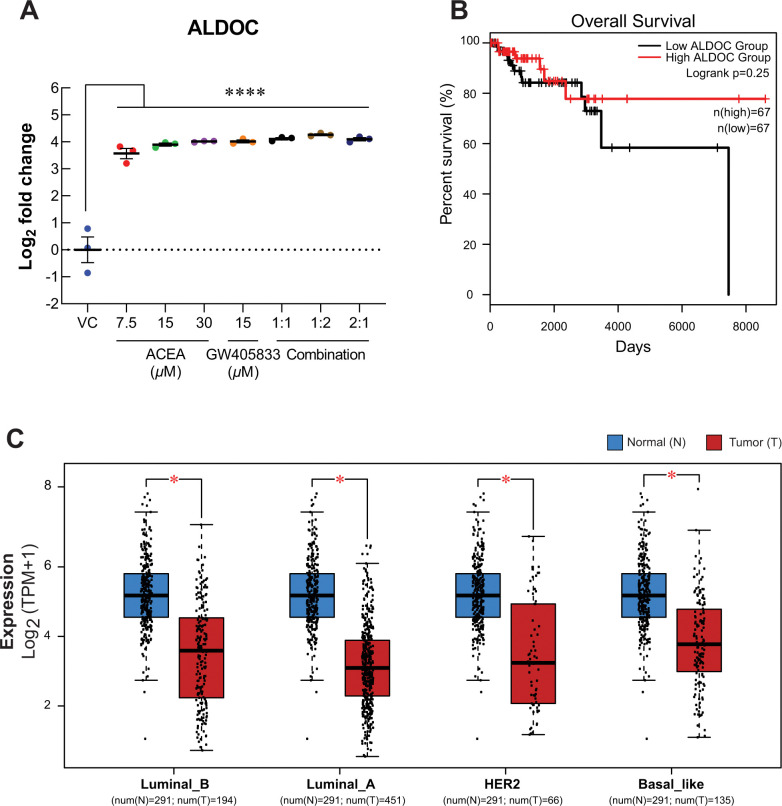
The common change of proteins after MDA-MB-231 was exposed to CB agonists was investigated. (A) The dot plot presented the level of ALDOC protein as log_2_ fold change normalized to control after 48 hours of treatment. (B) Overall survival analysis of ALDOC protein in triple-negative breast cancer (TNBC) (n = 67 in both groups). (C) Meta-analysis of ALDOC expression between normal tissues and breast cancer tissues. (*p < 0.05, ****p<0.0001).

Even though only 2:1 (ACEA:GW405833) combination significantly inhibited MDA-MB-231 colony formation after treatment withdrawal (Fig 1), individual and combination treatments were shown to suppress MDA-MB-231 cell viability and motility in our previous study [10] and Fig 6. We then investigated the common altered proteins in MDA-MB-231 cells exposed to CB agonists. There were 187 commonly altered proteins as compared to control from all treatments. Among these, our results showed that aldolase C (ALDOC) increased significantly in all conditions at least 11.85-fold (Fig 8A) as compared to control. The upregulation of ALDOC expression in MDA-MB-231 cells exposed to CB agonists was also confirmed by qRT-PCR as shown in S3I Fig. With this significant alteration, meta-analysis of ALDOC was performed to examine its association with breast cancer. Interestingly, by performing meta-analysis, the expression of ALDOC was downregulated in all breast cancer subtypes as shown in Fig 8C. Even though the results were not statistically significant, the increasing trend of percent survival in high ALDOC group was observed (Fig 8B). Since ALDOC downregulation was shown in all breast cancer subtypes, our results demonstrated the potential negative effect of CB agonist exposure on breast cancer via ALDOC induction.

## Discussions

With its important roles in various organs, different combinations between receptors and concentration of endocannabinoids presented in each microenvironment could affect how cells respond to the endocannabinoid system. Most common endocannabinoids including AEA and 2-AG were produced mainly by neurons in both peripheral and central nervous system; hence, these endocannabinoids could be found in many organs innervated by neurons [39]. Interestingly, many studies showed that ECS also affects several pathological conditions including cancer. ECS could regulate cancer progression via its immune modulation property as shown in bladder cancer and pancreatic ductal adenocarcinoma [40, 41]. Interestingly, several studies showed that endocannabinoid degrading enzymes were upregulated in cancer as compared to normal tissues, and their upregulation also correlated with cancer progression in many types of cancer including breast cancer as reviewed in [42, 43]. On the other hand, cancer cells also expressed CB1 and CB2 contributing to direct effects of endocannabinoids and CB agonists on cancer cell behaviors as reviewed in [44].

CB activation influenced variety of pathways in cancers, such as apoptosis, metastasis and angiogenesis [10, 45, 46]. For breast cancer, studies showed that breast cancer had elevated expression of CB1 and CB2, and the increased CB2 expression also associated with cancer aggressiveness [39, 44]. Exposing to CB agonists induced breast cancer cell death by apoptosis and ferroptosis [10, 47, 48]. Furthermore, CB activation could interfere with breast cancer bone metastasis via C-X-C motif chemokine ligand 12 (CXCL12) and C-X-C motif chemokine receptor 4 (CXCR4) pathway [49, 50]. Previous studies showed that MDA-MB-231, breast cancer cell line used in this study, also expressed both CB1 and CB2 [49, 51]. Previous study showed that MDA-MB-231 is more sensitive to CB agonists than the less aggressive cancer cell line, MCF-7. Furthermore, in this study also found that ACEA and GW405833 showed a higher cytotoxicity to MDA-MB-231 and 4T1 cells than less aggressive breast cancer, MCF-7 cells (S1 Fig). On the other hand, CB agonists were shown to have no significant effect on the non-cancerous breast cell line, MCF-10A, cell viability [52]. Similarly, our previous study showed that activation of CB2 on MDA-MB-231 not only suppressed breast cancer cell growth, but it also interfered with breast cancer and bone interaction. Moreover, while both CB1 and CB2 agonists inhibited breast cancer cell survival, combination of the two further enhanced this effect, especially the 2:1 (ACEA:GW405833) combination [10]. In this study, we demonstrated that the combination of CB1 agonist (ACEA) and CB2 agonist (GW405833) at 2:1 ratio also provided the long-term inhibitory effect on breast cancer colony formation even after treatment withdrawal. As cell cycle arrest could contribute to cell proliferation suppression, several studies showed that exposing to endocannabinoids, plant-derived cannabinoid or synthetic cannabinoid resulted in cell cycle arrest in gastric cancer, prostate cancer, renal cancer, hepatocellular carcinoma and breast cancer. Specifically, CBD treatment promoted G0/G1 phase and late S/G2 phase arrest in MDA-MB-231 [53]. Besides, exposing to AEA also induced S-phase cell cycle arrest in MDA-MB-231 resulting in reduction of cell population in G2/M-phase [54]. Herein, we demonstrated that co-administration of highly specific CB1 and CB2 agonists at a 2:1 ratio (ACEA: GW405833) caused S-phase arrest in MDA-MB-231. This could explain how ECS activation hindered breast cancer proliferation.

To further investigate the potential cell signaling alteration in breast cancer cells exposing to CB agonists and their combination, especially the 2:1 combination that showed the most profound inhibitory effects on breast cancer cells, proteomic analysis was performed. Our proteomic results revealed the alteration of several cell proliferation and cell cycle regulation-related proteins in breast cancer cells after 2:1 (CB1: CB2) agonist exposure. Our results showed that ZPR1, the proteins shown to be upregulated in breast cancer tissue and correlated with cancer progression, was significantly downregulated after CB agonist exposure [55]. Similarly, the presence of 2:1 combination of CB1:CB2 agonists also downregulated the expression of other proteins associated with cancer survival and proliferation including SHC1, MAPK15, RAN and TP53 [56–62]. On the other hand, upregulation of STAT1, MUC1 and CAV1 was found after treatment. These proteins were shown to suppress cell proliferation [63–65]. Likewise, the alteration in cell cycle control-related proteins was also found in breast cancer exposed to CB agonists. CDC20B, DCTN3, CEP295 and TIMLESS were shown to promote cell cycle progression and be associated with tumor progression in many types of cancer [66–77]. These proteins were significantly reduced corresponding to cell cycle arrest in breast cancer upon the exposure to 2:1 CB agonists as shown in this study. While other cell cycle-related proteins were downregulated in breast cancer cells exposed to CB agonists, ANAPC1 was upregulated in our study. ANAPC1 is one of anaphase-promoting complex (APC) subunits [78]. Generally, APC was known as a promoting factor for cell cycle progression. However, mutation of APC was found in many types of cancer, and APC downregulation was associated with the tumorigenesis and invasive phenotype of breast cancer [78, 79]. Therefore, APC upregulation with the exposure of CB agonists may negatively affect breast cancer progression corresponding to the phenotypes observed in this study. All of these alterations coincided with the reduced breast cancer cell growth upon the exposure CB agonists.

In the context of cell motility, prior investigations indicated that CB agonist exposure inhibited the metastatic characteristics of breast cancer both *in vitro* and *in vivo* [49, 80, 81]. Likewise, our group previously reported that ECS activation by CB1 or CB2 agonists significantly inhibited cell migration in MDA-MB-231 by wound-healing assay [10]. The results were also consistent in this study as CB agonists suppressed MDA-MB-231 cell invasion through extracellular matrix and lamellipodia formation. Moreover, specific combination of CB agonists (2:1; CB1:CB2 agonist) further promoted inhibitory effect of CB1 and CB2 agonists on cell invasion, which demonstrated the stronger effect of simultaneous CB agonist exposure as compared to the individual treatments. Corresponding to these effects, our proteomic analysis showed the significant alteration of several proteins associated with cell motility. Our results revealed the reduction in AXL expression, which was known as a regulator for epithelial-to-mesenchymal transition (EMT) of breast cancer [82]. AXL was shown to interact with several proteins contributing to cancer cell migration and invasion via RAC1 activation [83]. Even though the reduction was not significant in other conditions, RAC1 was notably decreased in MDA-MB-231 exposed to the 2:1 (CB1: CB2 agonists) combination where the most inhibitory effects were found. Several studies also indicated the involvement of VAV2 in RAC activation and cancer motility by functioning as RAC guanine exchange factor (GEF) to convert inactive RAC-GDP to the active RAC-GTP [37, 38, 84]. Our results revealed that VAV2 was also downregulated in all conditions in this study. Even though one of AXL interacting proteins, ELMO2, was found to be upregulated upon the exposure of CB agonists, the significantly decreased AXL, VAV2 and RAC1 as well as the diminished cell migration and invasion seen in previous and current studies indicated that negative effects of CB agonists on breast cancer cell motility might occur in ELMO2 independent pathway [10, 83]. Our data suggested the potential disruption of AXL-VAV2-RAC1 cell signaling pathway in MDA-MB-231 cells exposed to individual and combination of CB1 and CB2 agonists contributing to the decreased cell invasion ability.

One important characteristic of migratory cancer cells is lamellipodia formation, which is the formation of cellular protrusion resulting from actin re-organization [85, 86]. Rab5A was shown to promote lamellipodia formation and focal adhesion disassembly facilitating cell motility in RAC-dependent pathway [87–89]. Our results revealed the reduction of Rab5A in MDA-MB-231 breast cancer cells exposed to individual or the combination of CB agonists associated with the reduction in cell invasion and lamellipodia formation in this study. Since Rab5A was also reported to be associated with lymph node metastasis in breast cancer patients [90], the reduction of this protein upon CB agonist exposure suggested the potential obstruction of breast cancer metastasis in the presence of CB agonists. In addition to Rab5A, two proteins associated with cell motility, focal adhesion formation and cell-cell adhesion including ACTBL2 and CTNNA2 were also downregulated in breast cancer cells exposed to CB agonists [91, 92]. Taken together, our results suggested that the presence of CB agonists could potentially inhibit breast cancer cell invasion and lamellipodia formation by the downregulation of several cell locomotion-associated proteins. Mechanism wise, several proteins have been shown to be associated ERK-MAPK signaling pathways, such as RAC1 and Rab5 [93, 94].

Interestingly, we found that the exposure of CB agonists dramatically induced ALDOC expression of all conditions. While ALDOC is a crucial enzyme in glycolysis, many studies also showed its contribution in cell proliferation, cell morphology regulation and cancer progression in non-glycolytic pathways [95–97]. High level of ALDOC was found to correlate with the improved patient survival and reduced cancer progression in glioblastoma [98]. Moreover, the elevation of ALDOC in non-small cell lung cancer (NSCLC) promoted the expression of oncogenes through Wnt/β-catenin pathway [99]. Interestingly, the opposite trend was seen in breast cancer. Meta-analysis revealed that ALDOC expression was downregulated in all breast cancer subtypes, and the higher ALDOC expression was also associated with the higher survival of breast cancer patients. Similarly, previous study had showed that ALDOC overexpression was negatively correlated with aggressiveness of breast cancer [100]. Our results showed that ALDOC expression was dramatically increased in MDA-MB-231 exposed to CB agonists. This suggested another potential breast cancer suppression mechanism via ALDOC activation in breast cancer cells exposed to CB agonists. However, more studies are needed to verify the underlying mechanism for breast cancer suppression upon CB agonist exposure as well as the roles of ALDOC and its association with ECS in breast cancer progression.

## Conclusion

Our study demonstrated that the presence of CB agonists hindered breast cancer cell growth, cell cycle progression, invasion through extracellular matrices and lamellipodia formation. The exposure of specific combination of CB1 and CB2 agonists also enhanced their breast cancer suppression effects. Moreover, breast cancer survival and motility-related proteins affected by the presence of these agonists suggesting the potential pathways underlying their effects were also depicted in this study. Future study is still required to fully understand the role of ECS and the effects of CB agonist exposure in breast cancer progression.

## Supporting information

S1 Table The table shows the list of genes and their primers for gene expression study by qRT-PCR. (PDF)

S2 Table Unique protein alteration of MDA-MB-231 exposed to the combination treatment of ratio 2:1 (ACEA:GW405833). (PDF)

S1 Fig The effect of CB agonists on cell viability among different cell lines and their sensitivity to a specific 2:1 combination was investigated. The cytotoxic of ACEA on different cell lines (A) human triple-negative breast cancer; MDA-MB-231, (B) human luminal A breast cancer; MCF-7 and (C) mouse triple-negative breast cancer; 4T1. The cytotoxic of GW405833 on different cell lines (D) MDA-MB-231, (E) MCF-7 and (F) 4T1. The sensitivity comparison among different cell lines to (G) ACEA and (H) GW405833 were shown. (I) The cell viability was determined in three different cell lines under the exposure to a specific 2:1 combination of ACEA and GW405833. All experiments were done in three biological replicates with internal triplicates each. (*p<0.05 and ****p <0.0001 versus control).
(PDF)

S2 Fig A specific 2:1 combination of ACEA and GW405833 did not show a long-term inhibition on MCF-7 colony formation. A) Representative images of colony formation of MCF-7 pre-exposed to ACEA, GW405833 and their combination; (B) Colony number of MCF-7 pre-exposed to CB agonists was represented by mean ± SEM from three independent biological replicates with internal technical duplicates each. (**p <0.01, ***p <0.001, and ****p <0.0001).
(PDF)

S3 Fig Gene expression alterations of MDA-MB-231 cells exposed to CB agonists were investigated by qRT-PCR. (A) ZPR1, (B) SHC1, (C) MAPK15, (D) TP53, (E) ANAPC, (F) AXL, (G) VAV2, (H) RAC1 and (I) ALDOC. Bar graphs represented mean ± SEM from three independent biological replicates. (*p<0.05, **p<0.01 versus control, while #*p*<0.05 and ##*p*<0.01).
(PDF)

## References

[1] Bab I., Ofek O., Tam J., Rehnelt J., Zimmer A. Endocannabinoids and the regulation of bone metabolism. J Neuroendocrinol. 2008; 20 Suppl 1: 69–74. doi: 10.1111/j.1365-2826.2008.01675.x 18426503

[2] Hillard C.J. Biochemistry and pharmacology of the endocannabinoids arachidonylethanolamide and 2-arachidonylglycerol. Prostaglandins Other Lipid Mediat. 2000; 61(1–2): 3–18. doi: 10.1016/s0090-6980(00)00051-4 10785538

[3] Kohnz R.A., Nomura D.K. Chemical approaches to therapeutically target the metabolism and signaling of the endocannabinoid 2-AG and eicosanoids. Chem Soc Rev. 2014; 43(19): 6859–69. doi: 10.1039/c4cs00047a 24676249 PMC4159426

[4] Pacher P., Batkai S., Kunos G. The endocannabinoid system as an emerging target of pharmacotherapy. Pharmacol Rev. 2006; 58(3): 389–462. doi: 10.1124/pr.58.3.2 16968947 PMC2241751

[5] Howlett A.C. Abood M.E. CB(1) and CB(2) Receptor Pharmacology. Adv Pharmacol. 2017; 80: 169–206. doi: 10.1016/bs.apha.2017.03.007 28826534 PMC5812699

[6] Acharya N., Penukonda S., Shcheglova T., Hagymasi A.T., Basu S., Srivastava P.K. Endocannabinoid system acts as a regulator of immune homeostasis in the gut. Proc Natl Acad Sci U S A. 2017; 114(19): 5005–10. doi: 10.1073/pnas.1612177114 28439004 PMC5441729

[7] Ueda N., Tsuboi K., Uyama T., Ohnishi T. Biosynthesis and degradation of the endocannabinoid 2-arachidonoylglycerol. Biofactors. 2011; 37(1): 1–7. doi: 10.1002/biof.131 21328621

[8] Fraguas-Sanchez A.I., Martin-Sabroso C., Torres-Suarez A.I. Insights into the effects of the endocannabinoid system in cancer: a review. Br J Pharmacol. 2018; 175(13): 2566–80. doi: 10.1111/bph.14331 29663308 PMC6003657

[9] Hermanson D.J., Marnett L.J. Cannabinoids, endocannabinoids, and cancer. Cancer Metastasis Rev. 2011; 30(3–4): 599–612. doi: 10.1007/s10555-011-9318-8 22038019 PMC3366283

[10] Khunluck T., Lertsuwan K., Chutoe C., Sooksawanwit S., Inson I., Teerapornpuntakit J., et al. Activation of cannabinoid receptors in breast cancer cells improves osteoblast viability in cancer-bone interaction model while reducing breast cancer cell survival and migration. Sci Rep. 2022; 12(1): 7398. doi: 10.1038/s41598-022-11116-9 35513484 PMC9072415

[11] Lozano-Ondoua A.N., Hanlon K.E., Symons-Liguori A.M., Largent-Milnes T.M., Havelin J.J., Ferland H.L. 3rd, et al. Disease modification of breast cancer-induced bone remodeling by cannabinoid 2 receptor agonists. J Bone Miner Res. 2013; 28(1): 92–107. doi: 10.1002/jbmr.1732 22903605 PMC4745976

[12] Elbaz M., Ahirwar D., Ravi J., Nasser M.W., Ganju R.K. Novel role of cannabinoid receptor 2 in inhibiting EGF/EGFR and IGF-I/IGF-IR pathways in breast cancer. Oncotarget. 2017; 8(18): 29668–78. doi: 10.18632/oncotarget.9408 27213582 PMC5444694

[13] Hanlon K.E., Lozano-Ondoua A.N., Umaretiya P.J., Symons-Liguori A.M., Chandramouli A., Moy J.K., et al. Modulation of breast cancer cell viability by a cannabinoid receptor 2 agonist, JWH-015, is calcium dependent. Breast Cancer (Dove Med Press). 2016; 8: 59–71. doi: 10.2147/BCTT.S100393 27186076 PMC4847606

[14] Ahmad A. Pathways to breast cancer recurrence. ISRN Oncol. 2013; 2013: 290568. doi: 10.1155/2013/290568 23533807 PMC3603357

[15] Massague J., Obenauf A.C. Metastatic colonization by circulating tumour cells. Nature. 2016; 529(7586): 298–306. doi: 10.1038/nature17038 26791720 PMC5029466

[16] Rajendran V., Jain M.V. In Vitro Tumorigenic Assay: Colony Forming Assay for Cancer Stem Cells. Methods Mol Biol. 2018; 1692: 89–95. doi: 10.1007/978-1-4939-7401-6_8 28986889

[17] Karamanou K., Franchi M., Vynios D., Brezillon S. Epithelial-to-mesenchymal transition and invadopodia markers in breast cancer: Lumican a key regulator. Semin Cancer Biol. 2020; 62: 125–33. doi: 10.1016/j.semcancer.2019.08.003 31401293

[18] Lamouille S., Xu J., Derynck R. Molecular mechanisms of epithelial-mesenchymal transition. Nat Rev Mol Cell Biol. 2014; 15(3): 178–96. doi: 10.1038/nrm3758 24556840 PMC4240281

[19] Valastyan S., Weinberg R.A. Tumor metastasis: molecular insights and evolving paradigms. Cell. 2011; 147(2): 275–92. doi: 10.1016/j.cell.2011.09.024 22000009 PMC3261217

[20] Hillard C.J., Manna S., Greenberg M.J., Dicamelli R., Ross R.A., Stevenson L.A., et al. Synthesis and characterization of potent and selective agonists of the neuronal cannabinoid receptor (CB1). J Pharmacol Exp Ther. 1999; 289(3): 1427–33. 10336536

[21] Valenzano K.J., Tafesse L., Lee G., Harrison J.E., Boulet J.M., Gottshall S.L., et al. Pharmacological and pharmacokinetic characterization of the cannabinoid receptor 2 agonist, GW405833, utilizing rodent models of acute and chronic pain, anxiety, ataxia and catalepsy. Neuropharmacology. 2005; 48(5): 658–72. doi: 10.1016/j.neuropharm.2004.12.008 15814101

[22] Guzman C., Bagga M., Kaur A., Westermarck J., Abankwa D. ColonyArea: an ImageJ plugin to automatically quantify colony formation in clonogenic assays. PLoS One. 2014; 9(3): e92444. doi: 10.1371/journal.pone.0092444 24647355 PMC3960247

[23] Lertsuwan K., Nammultriputtar K., Nanthawuttiphan S., Tannop N., Teerapornpuntakit J., Thongbunchoo J., et al. Differential effects of Fe2+ and Fe3+ on osteoblasts and the effects of 1,25(OH)2D3, deferiprone and extracellular calcium on osteoblast viability under iron-overloaded conditions. PLoS One. 2020; 15(5): e0234009. doi: 10.1371/journal.pone.0234009 32470038 PMC7259719

[24] Krobthong S., Yingchutrakul Y., Samutrtai P., Hitakarun A., Siripattanapipong S., Leelayoova S., et al. Utilizing Quantitative Proteomics to Identify Species-Specific Protein Therapeutic Targets for the Treatment of Leishmaniasis. ACS Omega. 2022; 7(15): 12580–88. doi: 10.1021/acsomega.1c05792 35474788 PMC9026083

[25] Krobthong S., Yingchutrakul Y., Visessanguan W., Mahatnirunkul T., Samutrtai P., Chaichana C., et al. Study of the Lipolysis Effect of Nanoliposome-Encapsulated Ganoderma lucidum Protein Hydrolysates on Adipocyte Cells Using Proteomics Approach. Foods. 2021; 10(9. doi: 10.3390/foods10092157 34574267 PMC8468392

[26] Gillespie M., Jassal B., Stephan R., Milacic M., Rothfels K., Senff-Ribeiro A., et al. The reactome pathway knowledgebase 2022. Nucleic Acids Res. 2022; 50(D1): D687–D92. doi: 10.1093/nar/gkab1028 34788843 PMC8689983

[27] Thomas P.D., Ebert D., Muruganujan A., Mushayahama T., Albou L.P., Mi H. PANTHER: Making genome-scale phylogenetics accessible to all. Protein Sci. 2022; 31(1): 8–22. doi: 10.1002/pro.4218 34717010 PMC8740835

[28] Szklarczyk D., Gable A.L., Nastou K.C., Lyon D., Kirsch R., Pyysalo S., et al. The STRING database in 2021: customizable protein-protein networks, and functional characterization of user-uploaded gene/measurement sets. Nucleic Acids Res. 2021; 49(D1): D605–D12. doi: 10.1093/nar/gkaa1074 33237311 PMC7779004

[29] Shannon P., Markiel A., Ozier O., Baliga N.S., Wang J.T., Ramage D., et al. Cytoscape: a software environment for integrated models of biomolecular interaction networks. Genome Res. 2003; 13(11): 2498–504. doi: 10.1101/gr.1239303 14597658 PMC403769

[30] Tang Z., Kang B., Li C., Chen T., Zhang Z. GEPIA2: an enhanced web server for large-scale expression profiling and interactive analysis. Nucleic Acids Res. 2019; 47(W1): W556–W60. doi: 10.1093/nar/gkz430 31114875 PMC6602440

[31] Cancer Genome Atlas N. Comprehensive molecular portraits of human breast tumours. Nature. 2012; 490(7418): 61–70. doi: 10.1038/nature11412 23000897 PMC3465532

[32] Goldhirsch A., Winer E.P., Coates A.S., Gelber R.D., Piccart-Gebhart M., Thurlimann B., et al. Personalizing the treatment of women with early breast cancer: highlights of the St Gallen International Expert Consensus on the Primary Therapy of Early Breast Cancer 2013. Ann Oncol. 2013; 24(9): 2206–23. doi: 10.1093/annonc/mdt303 23917950 PMC3755334

[33] Pijuan J., Barcelo C., Moreno D.F., Maiques O., Siso P., Marti R.M., et al. In vitro Cell Migration, Invasion, and Adhesion Assays: From Cell Imaging to Data Analysis. Front Cell Dev Biol. 2019; 7: 107. doi: 10.3389/fcell.2019.00107 31259172 PMC6587234

[34] Weaver C.M., Makdissi S., Di Cara F. Modified protocol for culturing Drosophila S2 R+ cells and adult plasmatocytes to study actin cytoskeleton dynamics. STAR Protoc. 2022; 3(3): 101588. doi: 10.1016/j.xpro.2022.101588 35990744 PMC9389301

[35] Pothongsrisit S., Arunrungvichian K., Hayakawa Y., Sritularak B., Mangmool S., Pongrakhananon V. Erianthridin suppresses non-small-cell lung cancer cell metastasis through inhibition of Akt/mTOR/p70(S6K) signaling pathway. Sci Rep. 2021; 11(1): 6618. doi: 10.1038/s41598-021-85675-8 33758209 PMC7987990

[36] Shi H., Niimi A., Takeuchi T., Shiogama K., Mizutani Y., Kajino T., et al. CEBPgamma facilitates lamellipodia formation and cancer cell migration through CERS6 upregulation. Cancer Sci. 2021; 112(7): 2770–80. doi: 10.1111/cas.14928 33934437 PMC8253294

[37] Bid H.K., Roberts R.D., Manchanda P.K., Houghton P.J. RAC1: an emerging therapeutic option for targeting cancer angiogenesis and metastasis. Mol Cancer Ther. 2013; 12(10): 1925–34. doi: 10.1158/1535-7163.MCT-13-0164 24072884 PMC3823055

[38] Fukumoto Y., Kurita S., Takai Y., Ogita H. Role of scaffold protein afadin dilute domain-interacting protein (ADIP) in platelet-derived growth factor-induced cell movement by activating Rac protein through Vav2 protein. J Biol Chem. 2011; 286(50): 43537–48. doi: 10.1074/jbc.M111.308858 22027834 PMC3234878

[39] Pagano C., Navarra G., Coppola L., Bifulco M., Laezza C. Molecular Mechanism of Cannabinoids in Cancer Progression. Int J Mol Sci. 2021; 22(7. doi: 10.3390/ijms22073680 33916164 PMC8037087

[40] Gasperi V., Evangelista D., Oddi S., Florenzano F., Chiurchiu V., Avigliano L., et al. Regulation of inflammation and proliferation of human bladder carcinoma cells by type-1 and type-2 cannabinoid receptors. Life Sci. 2015; 138: 41–51. doi: 10.1016/j.lfs.2014.09.031 25445433

[41] Qiu C., Yang L., Wang B., Cui L., Li C., Zhuo Y., et al. The role of 2-arachidonoylglycerol in the regulation of the tumor-immune microenvironment in murine models of pancreatic cancer. Biomed Pharmacother. 2019; 115: 108952. doi: 10.1016/j.biopha.2019.108952 31078044

[42] Cherkasova V., Wang B., Gerasymchuk M., Fiselier A., Kovalchuk O., Kovalchuk I. Use of Cannabis and Cannabinoids for Treatment of Cancer. Cancers (Basel). 2022; 14(20. doi: 10.3390/cancers14205142 36291926 PMC9600568

[43] Qin H. Ruan Z.H. The role of monoacylglycerol lipase (MAGL) in the cancer progress. Cell Biochem Biophys. 2014; 70(1): 33–6. doi: 10.1007/s12013-014-9899-2 24633487

[44] Laezza C., Pagano C., Navarra G., Pastorino O., Proto M.C., Fiore D., et al. The Endocannabinoid System: A Target for Cancer Treatment. Int J Mol Sci. 2020; 21(3. doi: 10.3390/ijms21030747 31979368 PMC7037210

[45] Daris B., Tancer Verboten M., Knez Z., Ferk P. Cannabinoids in cancer treatment: Therapeutic potential and legislation. Bosn J Basic Med Sci. 2019; 19(1): 14–23. doi: 10.17305/bjbms.2018.3532 30172249 PMC6387667

[46] Vecera L., Gabrhelik T., Prasil P., Stourac P. The role of cannabinoids in the treatment of cancer. Bratisl Lek Listy. 2020; 121(1): 79–95. doi: 10.4149/BLL_2020_012 31950844

[47] Li P., Lin Q., Sun S., Yang N., Xia Y., Cao S., et al. Inhibition of cannabinoid receptor type 1 sensitizes triple-negative breast cancer cells to ferroptosis via regulating fatty acid metabolism. Cell Death Dis. 2022; 13(9): 808. doi: 10.1038/s41419-022-05242-5 36130940 PMC9492666

[48] Murase R., Kawamura R., Singer E., Pakdel A., Sarma P., Judkins J., et al. Targeting multiple cannabinoid anti-tumour pathways with a resorcinol derivative leads to inhibition of advanced stages of breast cancer. Br J Pharmacol. 2014; 171(19): 4464–77. doi: 10.1111/bph.12803 24910342 PMC4209152

[49] Nasser M.W., Qamri Z., Deol Y.S., Smith D., Shilo K., Zou X., et al. Crosstalk between chemokine receptor CXCR4 and cannabinoid receptor CB2 in modulating breast cancer growth and invasion. PLoS One. 2011; 6(9): e23901. doi: 10.1371/journal.pone.0023901 21915267 PMC3168464

[50] Sun Y., Mao X., Fan C., Liu C., Guo A., Guan S., et al. CXCL12-CXCR4 axis promotes the natural selection of breast cancer cell metastasis. Tumour Biol. 2014; 35(8): 7765–73. doi: 10.1007/s13277-014-1816-1 24810923 PMC4158177

[51] Qamri Z., Preet A., Nasser M.W., Bass C.E., Leone G., Barsky S.H., et al. Synthetic cannabinoid receptor agonists inhibit tumor growth and metastasis of breast cancer. Mol Cancer Ther. 2009; 8(11): 3117–29. doi: 10.1158/1535-7163.MCT-09-0448 19887554 PMC4128286

[52] Schoeman R., Beukes N., Frost C. Cannabinoid Combination Induces Cytoplasmic Vacuolation in MCF-7 Breast Cancer Cells. Molecules. 2020; 25(20. doi: 10.3390/molecules25204682 33066359 PMC7587381

[53] D’aloia A., Ceriani M., Tisi R., Stucchi S., Sacco E., Costa B. Cannabidiol Antiproliferative Effect in Triple-Negative Breast Cancer MDA-MB-231 Cells Is Modulated by Its Physical State and by IGF-1. Int J Mol Sci. 2022; 23(13. doi: 10.3390/ijms23137145 35806150 PMC9266539

[54] Laezza C., Pisanti S., Crescenzi E., Bifulco M. Anandamide inhibits Cdk2 and activates Chk1 leading to cell cycle arrest in human breast cancer cells. FEBS Lett. 2006; 580(26): 6076–82. doi: 10.1016/j.febslet.2006.09.074 17055492

[55] Liu B., Xing X., Li X., Guo Q., Xu T., Xu K. ZNF259 promotes breast cancer cells invasion and migration via ERK/GSK3beta/snail signaling. Cancer Manag Res. 2018; 10: 3159–68. doi: 10.2147/CMAR.S174745 30214308 PMC6126507

[56] Boudhraa Z., Carmona E., Provencher D., Mes-Masson A.M. Ran GTPase: A Key Player in Tumor Progression and Metastasis. Front Cell Dev Biol. 2020; 8: 345. doi: 10.3389/fcell.2020.00345 32528950 PMC7264121

[57] Chen J., Gao G., Li L., Ding J., Chen X., Lei J., et al. Pan-Cancer Study of SHC-Adaptor Protein 1 (SHC1) as a Diagnostic, Prognostic and Immunological Biomarker in Human Cancer. Front Genet. 2022; 13: 817118. doi: 10.3389/fgene.2022.817118 35601500 PMC9115805

[58] Gasco M., Shami S., Crook T. The p53 pathway in breast cancer. Breast Cancer Res. 2002; 4(2): 70–6. doi: 10.1186/bcr426 11879567 PMC138723

[59] Jin D.H., Lee J., Kim K.M., Kim S., Kim D.H., Park J. Overexpression of MAPK15 in gastric cancer is associated with copy number gain and contributes to the stability of c-Jun. Oncotarget. 2015; 6(24): 20190–203. doi: 10.18632/oncotarget.4171 26035356 PMC4652997

[60] Li X., Niu Z., Sun C., Zhuo S., Yang H., Yang X., et al. Regulation of P53 signaling in breast cancer by the E3 ubiquitin ligase RNF187. Cell Death Dis. 2022; 13(2): 149. doi: 10.1038/s41419-022-04604-3 35165289 PMC8844070

[61] Li Z., Li N., Shen L., Fu J. Quantitative Proteomic Analysis Identifies MAPK15 as a Potential Regulator of Radioresistance in Nasopharyngeal Carcinoma Cells. Front Oncol. 2018; 8: 548. doi: 10.3389/fonc.2018.00548 30524968 PMC6262088

[62] Wright K.D., Miller B.S., El-Meanawy S., Tsaih S.W., Banerjee A., Geurts A.M., et al. The p52 isoform of SHC1 is a key driver of breast cancer initiation. Breast Cancer Res. 2019; 21(1): 74. doi: 10.1186/s13058-019-1155-7 31202267 PMC6570928

[63] Koromilas A.E., Sexl V. The tumor suppressor function of STAT1 in breast cancer. JAKSTAT. 2013; 2(2): e23353. doi: 10.4161/jkst.23353 24058806 PMC3710319

[64] Lillehoj E.P., Lu W., Kiser T., Goldblum S.E., Kim K.C. MUC1 inhibits cell proliferation by a beta-catenin-dependent mechanism. Biochim Biophys Acta. 2007; 1773(7): 1028–38. doi: 10.1016/j.bbamcr.2007.04.009 17524503 PMC2349984

[65] Yang J., Zhu T., Zhao R., Gao D., Cui Y., Wang K., et al. Caveolin-1 Inhibits Proliferation, Migration, and Invasion of Human Colorectal Cancer Cells by Suppressing Phosphorylation of Epidermal Growth Factor Receptor. Med Sci Monit. 2018; 24: 332–41. doi: 10.12659/msm.907782 29339715 PMC5783188

[66] Bruno S., Ghelli Luserna Di Rora A., Napolitano R., Soverini S., Martinelli G.,Simonetti G. CDC20 in and out of mitosis: a prognostic factor and therapeutic target in hematological malignancies. J Exp Clin Cancer Res. 2022; 41(1): 159. doi: 10.1186/s13046-022-02363-9 35490245 PMC9055704

[67] Chi L., Zou Y., Qin L., Ma W., Hao Y., Tang Y., et al. TIMELESS contributes to the progression of breast cancer through activation of MYC. Breast Cancer Res. 2017; 19(1): 53. doi: 10.1186/s13058-017-0838-1 28464854 PMC5414141

[68] Mcfarlane R.J., Mian S., Dalgaard J.Z. The many facets of the Tim-Tipin protein families’ roles in chromosome biology. Cell Cycle. 2010; 9(4): 700–5. doi: 10.4161/cc.9.4.10676 20139726

[69] Neilsen B.K., Frodyma D.E., Mccall J.L., Fisher K.W., Lewis R.E. ERK-mediated TIMELESS expression suppresses G2/M arrest in colon cancer cells. PLoS One. 2019; 14(1): e0209224. doi: 10.1371/journal.pone.0209224 30629587 PMC6328106

[70] Quintyne N.J., Schroer T.A. Distinct cell cycle-dependent roles for dynactin and dynein at centrosomes. J Cell Biol. 2002; 159(2): 245–54. doi: 10.1083/jcb.200203089 12391026 PMC2173046

[71] Revinski D.R., Zaragosi L.E., Boutin C., Ruiz-Garcia S., Deprez M., Thome V., et al. CDC20B is required for deuterosome-mediated centriole production in multiciliated cells. Nat Commun. 2018; 9(1): 4668. doi: 10.1038/s41467-018-06768-z 30405130 PMC6220262

[72] Smith-Roe S.L., Patel S.S., Simpson D.A., Zhou Y.C., Rao S., Ibrahim J.G., et al. Timeless functions independently of the Tim-Tipin complex to promote sister chromatid cohesion in normal human fibroblasts. Cell Cycle. 2011; 10(10): 1618–24. doi: 10.4161/cc.10.10.15613 21508667 PMC3127161

[73] Su X., Li H., Chen S., Qin C. Study on the Prognostic Values of Dynactin Genes in Low-Grade Glioma. Technol Cancer Res Treat. 2021; 20: 15330338211010143. doi: 10.1177/15330338211010143 33896271 PMC8085377

[74] Tsuchiya Y., Yoshiba S., Gupta A., Watanabe K., Kitagawa D. Cep295 is a conserved scaffold protein required for generation of a bona fide mother centriole. Nat Commun. 2016; 7: 12567. doi: 10.1038/ncomms12567 27562453 PMC5007451

[75] Vangenderen C., Harkness T.a.A.,Arnason T.G. The role of Anaphase Promoting Complex activation, inhibition and substrates in cancer development and progression. Aging (Albany NY). 2020; 12(15): 15818–55. doi: 10.18632/aging.103792 32805721 PMC7467358

[76] Zhou J., Chen C., Zhao X., Jiang T., Jiang Y., Dai J., et al. Coding variants in the PCNT and CEP295 genes contribute to breast cancer risk in Chinese women. Pathol Res Pract. 2021; 225: 153581. doi: 10.1016/j.prp.2021.153581 34418690

[77] Zhou J., Zhang Y., Zou X., Kuai L., Wang L., Wang J., et al. Aberrantly Expressed Timeless Regulates Cell Proliferation and Cisplatin Efficacy in Cervical Cancer. Hum Gene Ther. 2020; 31(5–6): 385–95. doi: 10.1089/hum.2019.080 31870179

[78] Melloy P.G. The anaphase-promoting complex: A key mitotic regulator associated with somatic mutations occurring in cancer. Genes Chromosomes Cancer. 2020; 59(3): 189–202. doi: 10.1002/gcc.22820 31652364

[79] Park K.H., Choi S.E., Eom M., Kang Y. Downregulation of the anaphase-promoting complex (APC)7 in invasive ductal carcinomas of the breast and its clinicopathologic relationships. Breast Cancer Res. 2005; 7(2): R238–47. doi: 10.1186/bcr978 15743504 PMC1064132

[80] Caffarel M.M., Andradas C., Mira E., Perez-Gomez E., Cerutti C., Moreno-Bueno G., et al. Cannabinoids reduce ErbB2-driven breast cancer progression through Akt inhibition. Mol Cancer. 2010; 9: 196. doi: 10.1186/1476-4598-9-196 20649976 PMC2917429

[81] Elbaz M., Nasser M.W., Ravi J., Wani N.A., Ahirwar D.K., Zhao H., et al. Modulation of the tumor microenvironment and inhibition of EGF/EGFR pathway: novel anti-tumor mechanisms of Cannabidiol in breast cancer. Mol Oncol. 2015; 9(4): 906–19. doi: 10.1016/j.molonc.2014.12.010 25660577 PMC4387115

[82] Gjerdrum C., Tiron C., Hoiby T., Stefansson I., Haugen H., Sandal T., et al. Axl is an essential epithelial-to-mesenchymal transition-induced regulator of breast cancer metastasis and patient survival. Proc Natl Acad Sci U S A. 2010; 107(3): 1124–9. doi: 10.1073/pnas.0909333107 20080645 PMC2824310

[83] Li W., Xiong X., Abdalla A., Alejo S., Zhu L., Lu F., et al. HGF-induced formation of the MET-AXL-ELMO2-DOCK180 complex promotes RAC1 activation, receptor clustering, and cancer cell migration and invasion. J Biol Chem. 2018; 293(40): 15397–418. doi: 10.1074/jbc.RA118.003063 30108175 PMC6177597

[84] Menard L., Parker P.J., Kermorgant S. Receptor tyrosine kinase c-Met controls the cytoskeleton from different endosomes via different pathways. Nat Commun. 2014; 5: 3907. doi: 10.1038/ncomms4907 24835487

[85] Machesky L.M. Lamellipodia and filopodia in metastasis and invasion. FEBS Lett. 2008; 582(14): 2102–11. doi: 10.1016/j.febslet.2008.03.039 18396168

[86] Yamaguchi H., Condeelis J. Regulation of the actin cytoskeleton in cancer cell migration and invasion. Biochim Biophys Acta. 2007; 1773(5): 642–52. doi: 10.1016/j.bbamcr.2006.07.001 16926057 PMC4266238

[87] Mendoza P., Ortiz R., Diaz J., Quest A.F., Leyton L., Stupack D., et al. Rab5 activation promotes focal adhesion disassembly, migration and invasiveness in tumor cells. J Cell Sci. 2013; 126(Pt 17): 3835–47. doi: 10.1242/jcs.119727 23813952 PMC4074302

[88] Spaargaren M., Bos J.L. Rab5 induces Rac-independent lamellipodia formation and cell migration. Mol Biol Cell. 1999; 10(10): 3239–50. doi: 10.1091/mbc.10.10.3239 10512863 PMC25585

[89] Zech T., Machesky L. Rab5 and rac team up in cell motility. Cell. 2008; 134(1): 18–20. doi: 10.1016/j.cell.2008.06.039 18614005

[90] Yang P.S., Yin P.H., Tseng L.M., Yang C.H., Hsu C.Y., Lee M.Y., et al. Rab5A is associated with axillary lymph node metastasis in breast cancer patients. Cancer Sci. 2011; 102(12): 2172–8. doi: 10.1111/j.1349-7006.2011.02089.x 21895870

[91] Fanjul-Fernandez M., Quesada V., Cabanillas R., Cadinanos J., Fontanil T., Obaya A., et al. Cell-cell adhesion genes CTNNA2 and CTNNA3 are tumour suppressors frequently mutated in laryngeal carcinomas. Nat Commun. 2013; 4: 2531. doi: 10.1038/ncomms3531 24100690

[92] Malek N., Michrowska A., Mazurkiewicz E., Mrowczynska E., Mackiewicz P., Mazur A.J. The origin of the expressed retrotransposed gene ACTBL2 and its influence on human melanoma cells’ motility and focal adhesion formation. Sci Rep. 2021; 11(1): 3329. doi: 10.1038/s41598-021-82074-x 33558623 PMC7870945

[93] Germann U.A. Alam J.J. P38alpha MAPK Signaling-A Robust Therapeutic Target for Rab5-Mediated Neurodegenerative Disease. Int J Mol Sci. 2020; 21(15. doi: 10.3390/ijms21155485 32751991 PMC7432772

[94] Vial E., Sahai E., Marshall C.J. ERK-MAPK signaling coordinately regulates activity of Rac1 and RhoA for tumor cell motility. Cancer Cell. 2003; 4(1): 67–79. doi: 10.1016/s1535-6108(03)00162-4 12892714

[95] Caspi M., Perry G., Skalka N., Meisel S., Firsow A., Amit M., et al. Aldolase positively regulates of the canonical Wnt signaling pathway. Mol Cancer. 2014; 13: 164. doi: 10.1186/1476-4598-13-164 24993527 PMC4094682

[96] Merkulova M., Hurtado-Lorenzo A., Hosokawa H., Zhuang Z., Brown D., Ausiello D.A., et al. Aldolase directly interacts with ARNO and modulates cell morphology and acidic vesicle distribution. Am J Physiol Cell Physiol. 2011; 300(6): C1442–55. doi: 10.1152/ajpcell.00076.2010 21307348 PMC3118619

[97] Ritterson Lew C., Tolan D.R. Targeting of several glycolytic enzymes using RNA interference reveals aldolase affects cancer cell proliferation through a non-glycolytic mechanism. J Biol Chem. 2012; 287(51): 42554–63. doi: 10.1074/jbc.M112.405969 23093405 PMC3522257

[98] Chang Y.C., Tsai H.F., Huang S.P., Chen C.L., Hsiao M., Tsai W.C. Enrichment of Aldolase C Correlates with Low Non-Mutated IDH1 Expression and Predicts a Favorable Prognosis in Glioblastomas. Cancers (Basel). 2019; 11(9. doi: 10.3390/cancers11091238 31450822 PMC6770576

[99] Yuan J., Xing H., Li Y., Song Y., Zhang N., Xie M., et al. EPB41 suppresses the Wnt/beta-catenin signaling in non-small cell lung cancer by sponging ALDOC. Cancer Lett. 2021; 499: 255–64. doi: 10.1016/j.canlet.2020.11.024 33242559

[100] Reinsborough C.W., Ipas H., Abell N.S., Gouws E.B., Williams J.P., Mercado M., et al. BCDIN3D RNA methyltransferase stimulates Aldolase C expression and glycolysis through let-7 microRNA in breast cancer cells. Oncogene. 2021; 40(13): 2395–406. doi: 10.1038/s41388-021-01702-y 33664453 PMC8026734

