# Supplementary material for: Combinatorial effects of cannabinoid receptor 1 and 2 agonists on characteristics and proteomic alteration in MDA-MB-231 breast cancer cells
Source: PLoS One. 2024 Nov 11;19(11):e0312851. doi: 10.1371/journal.pone.0312851 (PMC11554208; doi:10.1371/journal.pone.0312851)
Supplement: S1 Table — (PDF) [file pone.0312851.s001.pdf]

S1 Table. The table shows the list of genes and their primers for gene expression study by qRT-PCR.

Gene	Accession number	Primer sequences
**ZPR1**<br>(ZPR1 zinc finger)	NM_003904.5	***Forward***<br>5’-CACCAAGATTCCCTTCTTCA-3’<br>***Reverse***<br>5’-CTGACAGACAAAGTGTAGCG-3’
**SHC1**<br>(SHC adaptor protein 1)	XM_054338223.1	***Forward***<br>5’-CAGGCAGAGAGCTTTTTGAT-3’<br>***Reverse***<br>5’-CCATTGATAGCAGGATTGGG-3’
**MAPK15**<br>(mitogen-activated protein kinase 15)	NM_139021.3	***Forward***<br>5’-GCATTGTCCGGAGATACCTA-3’<br>***Reverse***<br>5’-GGGCATCTGTCTTATCCCTA-3’
**TP53**<br>(Tumor protein p53)	NM_000546.6	***Forward***<br>5’-GGACACTTTGCGTTCGG-3’<br>***Reverse***<br>5’-GCTAGGATCTGACTGCGG-3’
**ANAPC1**<br>(Anaphase promoting complex subunit 1)	NM_022662.4	***Forward***<br>5’-CAGAAGCTGTCTGTCTCTTG-3’<br>***Reverse***<br>5’-GGAACATCTGATGAGAGCAC-3’
**AXL**<br>(AXL receptor tyrosine kinase)	NM_001278599.2	***Forward***<br>5’-CTCTCAGGATCCAAGCTAAG-3’<br>***Reverse***<br>5’-TGGGATAGGTAGGAAGACAG-3’
**VAV2**<br>(VAV guanine nucleotide exchange factor 2)	NM_001134398.2	***Forward***<br>5’-TACAGCTACGAGCTCAAGGA-3’<br>***Reverse***<br>5’-CACATTTTCCCGTGAGACTTC-3’
**RAC1**<br>(Rac family small GTPase 1)	NM_006908.5	***Forward***<br>5’-GGTAGATGGAAAACCGGTGA-3’<br>***Reverse***<br>5’-GAACACATCTGTTTGCGGAT-3’
**ALDOC**<br>(Aldolase C)	NM_005165.3	***Forward***<br>5’-CATAGGATGGGAGGATAGGG-3’<br>***Reverse***<br>5’-TCATGGGAAAATTGTGGGAG-3’
**ACTB**<br>(β-actin; housekeeping)	NM_001101.5	***Forward***<br>5’-AAAACTGGAACGGTGAAGG-3’<br>***Reverse***<br>5’-ACAACGCATCTCATATTTGGAA-3’
