# Supplementary material for: Combinatorial effects of cannabinoid receptor 1 and 2 agonists on characteristics and proteomic alteration in MDA-MB-231 breast cancer cells
Source: PLoS One. 2024 Nov 11;19(11):e0312851. doi: 10.1371/journal.pone.0312851 (PMC11554208; doi:10.1371/journal.pone.0312851)
Supplement: S2 Table — (PDF) [file pone.0312851.s002.pdf]

S2 Table. Unique protein alteration of MDA-MB-231 exposed to the combination treatment of ratio 2:1 (ACEA:GW405833) for 48 hours, were identified.

Accession	Log$_2$ intensities						-Log (*p-value*)	Direction
	Vehicle control			Combination 2:1				
	Rep.1	Rep.2	Rep.3	Rep.1	Rep.2	Rep.3		
**OGN**	19.257	18.855	18.407	17.955	18.014	17.815	2.033	Down
**P4HA1**	20.999	21.495	21.782	22.351	22.238	22.299	2.102	Up
**EIF3H**	19.948	20.364	20.142	18.948	19.427	19.540	2.150	Down
**RRM2**	18.095	17.715	18.292	18.684	18.703	18.772	2.162	Up
**ZPR1**	20.363	20.386	20.435	19.499	19.836	19.975	2.279	Down
**ANAPC1**	15.418	15.368	15.827	16.160	16.308	16.192	2.337	Up
**LRP10**	16.694	16.718	16.411	17.231	17.057	17.348	2.359	Up
**MRPL1**	19.692	19.525	19.670	18.878	19.215	18.835	2.476	Down
**HEBP1**	19.972	19.885	20.185	20.721	20.571	20.513	2.561	Up
**PHYHD1**	19.547	19.477	19.431	19.003	18.978	18.695	2.632	Down
**SCARB2**	19.623	19.262	19.311	20.017	20.046	20.114	2.662	Up
**CD81**	20.372	20.558	20.595	21.189	21.045	21.365	2.753	Up
**BROX**	20.050	20.030	19.976	19.586	19.408	19.296	2.835	Down
**SMG9**	17.606	17.668	17.412	16.888	16.965	16.716	2.885	Down
**LNPEP**	20.079	19.918	19.821	20.544	20.465	20.567	2.898	Up
**ABCD3**	19.837	19.857	20.077	20.505	20.625	20.497	2.909	Up
**ZNF207**	19.669	19.616	19.702	19.126	18.898	18.773	2.942	Down
**PRPF3**	17.556	17.810	17.806	16.820	17.085	16.933	2.962	Down
**EHD2**	21.597	21.373	21.396	22.114	22.021	22.129	3.071	Up
**MT-CYB**	18.332	18.348	18.374	17.883	17.748	17.665	3.134	Down
**DICER1**	17.860	17.557	17.815	16.799	16.973	16.903	3.188	Down
**ATP1B3**	20.405	20.522	20.426	21.221	21.108	21.005	3.219	Up
**AK2**	21.823	21.783	21.652	21.160	21.097	21.182	3.284	Down
**SEC61A1**	21.671	21.753	21.762	20.958	20.996	21.164	3.310	Down
**NUP107**	19.129	19.121	19.010	19.856	19.701	19.749	3.425	Up
**SLC25A22**	19.257	18.855	18.407	17.955	18.014	17.815	2.033	Down
