# Supplementary material for: Combinatorial effects of cannabinoid receptor 1 and 2 agonists on characteristics and proteomic alteration in MDA-MB-231 breast cancer cells
Source: PLoS One. 2024 Nov 11;19(11):e0312851. doi: 10.1371/journal.pone.0312851 (PMC11554208; doi:10.1371/journal.pone.0312851)
Supplement: S1 Fig — The cytotoxic of ACEA on different cell lines (A) human triple-negative breast cancer; MDA-MB-231, (B) human luminal A breast cancer; MCF-7 and (C) mouse triple-negative breast cancer; 4T1. The cytotoxic of GW405833 on different cell lines (D) MDA-MB-231, (E) MCF-7 and (F) 4T1. The sensitivity comparison among different cell lines to (G) ACEA and (H) GW405833 were shown. (I) The cell viability was determined in three different cell lines under the exposure to a specific 2:1 combination of ACEA and GW405833. All experiments were done in three biological replicates with internal triplicates each. (*p<0.05 and ****p <0.0001 versus control). (PDF) [file pone.0312851.s003.pdf]

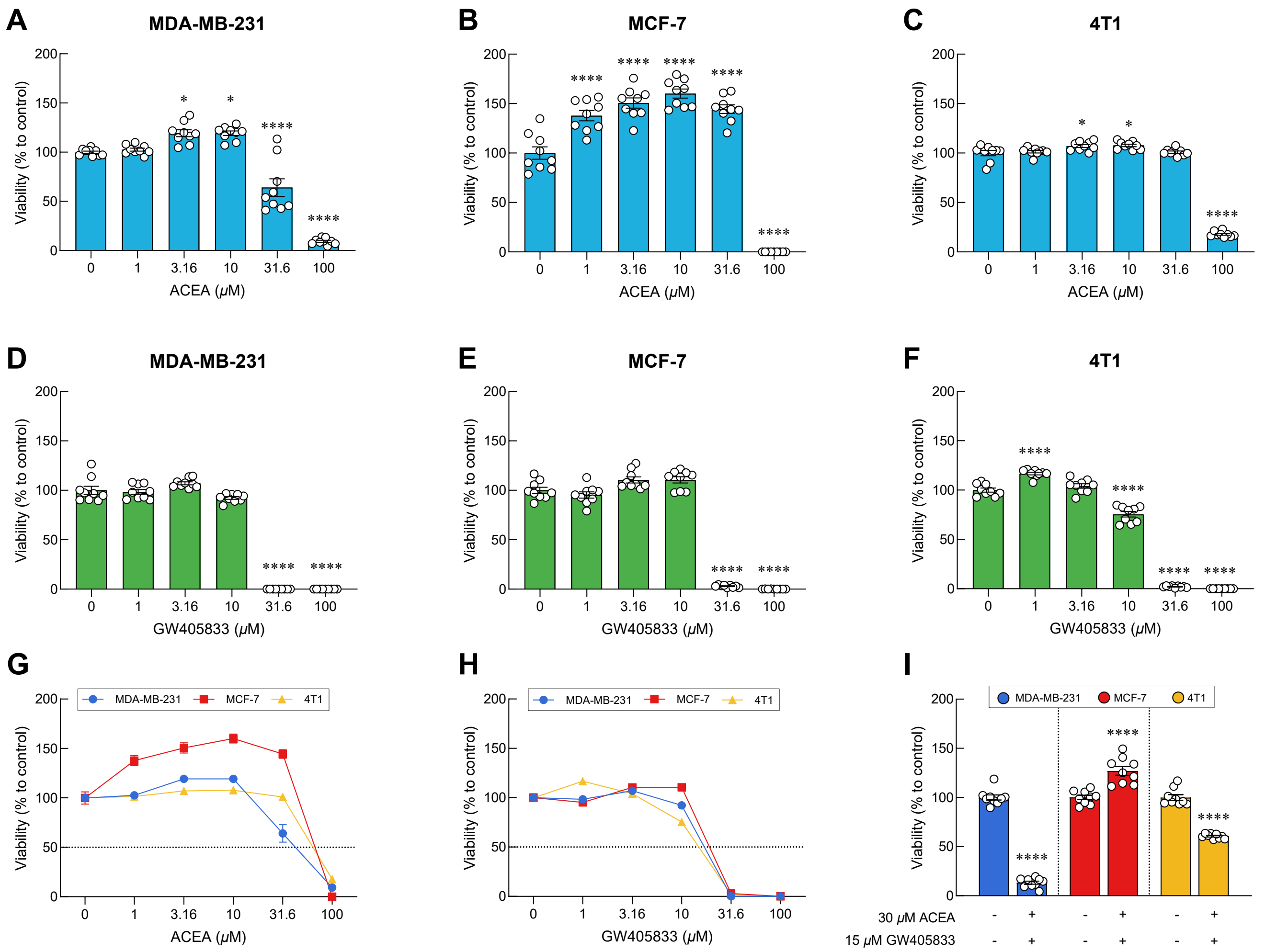

Cell lines	IC_{50} (μM)
MDA-MB-231	38.08
MCF-7	80.74
4T1	88.09

Cell lines	IC_{50} (μM)
MDA-MB-231	17.24
MCF-7	21.65
4T1	15.08

S1 Fig. The effect of CB agonists on cell viability among different cell lines and their sensitivity to a specific 2:1 combination was investigated. The cytotoxic of ACEA on different cell lines (A) human triple-negative breast cancer; MDA-MB-231, (B) human luminal A breast cancer; MCF-7 and (C) mouse triple-negative breast cancer; 4T1. The cytotoxic of GW405833 on different cell lines (D) MDA-MB-231, (E) MCF-7 and (F) 4T1. The sensitivity comparison among different cell lines to (G) ACEA and (H) GW405833 were shown. (I) The cell viability was determined in three different cell lines under the exposure to a specific 2:1 combination of ACEA and GW405833. All experiment were done in three biological replicates with internal triplicates each. (**p*<0.05 and *****p* <0.0001 versus control)
