# Supplementary material for: Combinatorial effects of cannabinoid receptor 1 and 2 agonists on characteristics and proteomic alteration in MDA-MB-231 breast cancer cells
Source: PLoS One. 2024 Nov 11;19(11):e0312851. doi: 10.1371/journal.pone.0312851 (PMC11554208; doi:10.1371/journal.pone.0312851)
Supplement: S2 Fig — A) Representative images of colony formation of MCF-7 pre-exposed to ACEA, GW405833 and their combination; (B) Colony number of MCF-7 pre-exposed to CB agonists was represented by mean ± SEM from three independent biological replicates with internal technical duplicates each. (**p <0.01, ***p <0.001, and ****p <0.0001). (PDF) [file pone.0312851.s004.pdf]

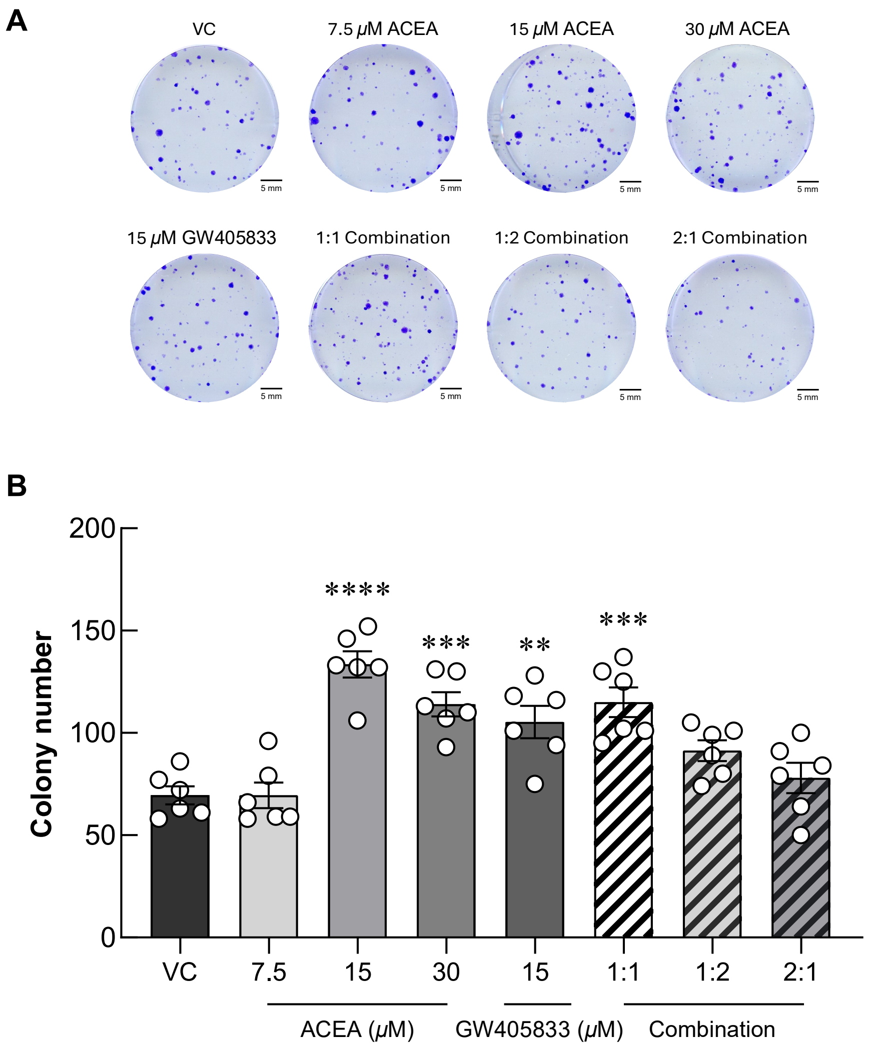

S2 Fig. A specific 2:1 combination of ACEA and GW405833 did not show a long-term inhibition on MCF-7 colony formation. (A) Representative images of colony formation of MCF-7 pre-exposed to ACEA, GW405833 and their combination; (B) Colony number of MCF-7 pre-exposed to CB agonists was represented by mean ± SEM from three independent biological replicates with internal technical duplicates each. (***p* < 0.01, ****p* <0.001, and *****p* <0.0001 versus control)
