# Supplementary material for: Combinatorial effects of cannabinoid receptor 1 and 2 agonists on characteristics and proteomic alteration in MDA-MB-231 breast cancer cells
Source: PLoS One. 2024 Nov 11;19(11):e0312851. doi: 10.1371/journal.pone.0312851 (PMC11554208; doi:10.1371/journal.pone.0312851)
Supplement: S3 Fig — (A) ZPR1, (B) SHC1, (C) MAPK15, (D) TP53, (E) ANAPC, (F) AXL, (G) VAV2, (H) RAC1 and (I) ALDOC. Bar graphs represented mean ± SEM from three independent biological replicates. (*p<0.05, **p<0.01 versus control, while #p<0.05 and ##p<0.01). (PDF) [file pone.0312851.s005.pdf]

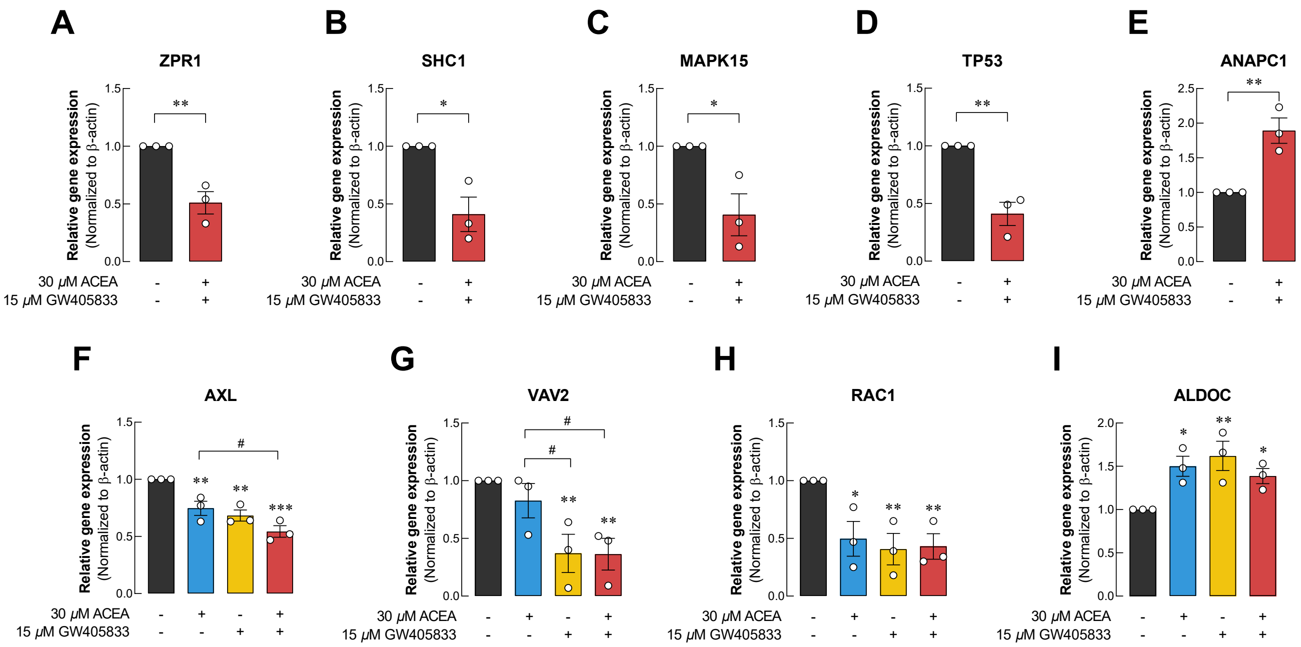

S3 Fig. Gene expression alterations of MDA-MB-231 cells exposed to CB agonists were investigated by qRT-PCR. (A) ZPR1, (B) SHC1, (C) MAPK15, (D) TP53, (E) ANAPC, (F) AXL, (G) VAV2, (H) RAC1 and (I) ALDOC. Bar graphs represented mean ± SEM from three independent biological replicates. (**p*<0.05, ***p*<0.01 versus control, while #*p*<0.05 and ##*p*<0.01)
